# Design of Novel Relaxase Substrates Based on Rolling Circle Replicases for Bioconjugation to DNA Nanostructures

**DOI:** 10.1371/journal.pone.0152666

**Published:** 2016-03-30

**Authors:** Sandra Sagredo, Fernando de la Cruz, Gabriel Moncalián

**Affiliations:** Departamento de Biología Molecular e Instituto de Biomedicina y Biotecnología de Cantabria, Universidad de Cantabria-Consejo Superior de Investigaciones Científicas-SODERCAN, C/ Albert Einstein 22, 39011, Santander, Spain; Centre National de la Recherche Scientifique, Aix-Marseille Université, FRANCE

## Abstract

During bacterial conjugation and rolling circle replication, HUH endonucleases, respectively known as relaxases and replicases, form a covalent bond with ssDNA when they cleave their target sequence (*nic* site). Both protein families show structural similarity but limited amino acid identity. Moreover, the organization of the inverted repeat (IR) and the loop that shape the *nic* site differs in both proteins. Arguably, replicases cleave their target site more efficiently, while relaxases exert more biochemical control over the process. Here we show that engineering a relaxase target by mimicking the replicase target, results in enhanced formation of protein-DNA covalent complexes. Three widely different relaxases, which belong to MOB_F_, MOB_Q_ and MOB_P_ families, can properly cleave DNA sequences with permuted target sequences. Collaterally, the secondary structure that the permuted targets acquired within a supercoiled plasmid DNA resulted in poor conjugation frequencies underlying the importance of relaxase accessory proteins in conjugative DNA processing. Our results reveal that relaxase and replicase targets can be interchangeable *in vitro*. The new Rep substrates provide new bioconjugation tools for the design of sophisticated DNA-protein nanostructures.

## Introduction

HUH proteins are enzymes widespread in all three domains of life, where they process DNA during initiation of rolling circle replication (RCR) of certain phages and eukaryotic viruses, conjugative transfer of plasmid between cells and transposition of insertion sequences and helitrons [1]. These enzymes contain an HUH motif, in which two conserved histidines (H) involved in metal coordination are separated by a hydrophobic residue (U). HUH endonucleases also contain a Y motif, which contains the tyrosine(s) involved in the nucleophilic attack to cleave and rejoin the target single strand DNA (ssDNA). One attractive feature of HUH proteins is that the nucleophilic attack on specific ssDNA sequences results in stable protein-DNA covalent linkages. Thus, HUH proteins provide a tool for site specific bioconjugation of proteins to ssDNA, for instance to DNA origami nanostructures, where ssDNA is folded into a desired shape with the aid of hundreds of oligonucleotides named “staples” [2]. Relaxases such as TrwC of plasmid R388 or TraI of plasmid F have been proven to form protein-DNA conjugates efficiently [3]. Using this approach, DNA can be conjugated to any desired protein fused to either the N-terminus or the C-terminus of recombinant relaxases, without losing activity. Moreover, HUH proteins exhibit site-specific recombination and have been used as an efficient strategy for genome editing. The replicase of Adeno-associate virus (AAV) and relaxase TrwC from plasmid R388 catalyze site-specific DNA integration into human genomes [4] and thus constitute potential new tools for genome editing.

**Fig 1:**
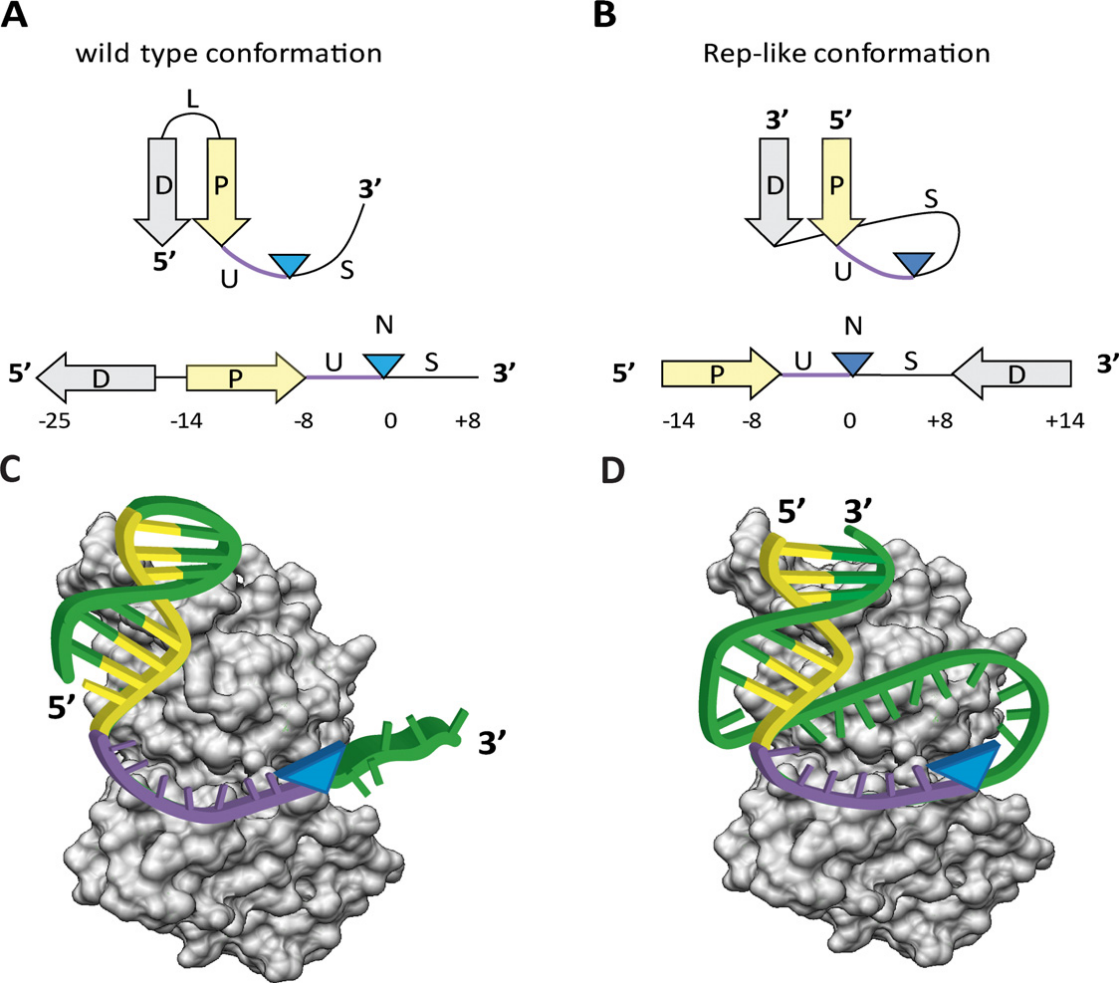
Scheme of relaxase and replicase DNA targets. (A) The relaxase DNA substrate contains an IR, defined by a Distal arm (D) and a Proximal Arm (P), that shape a hairpin structure. The *nic* site (N) is located between a U-turn (U) sequence and a ssDNA strand (S) that is tethered to the relaxase after cleavage. (B) The DNA substrate cleaved by replicases has the Distal arm (D) located downstream from *nic* (N), which allows stem-loop formation. Rep-like substrates for relaxases were designed by displacing the D sequence to the 3’ end of S of their original wt substrate. (C) Scheme depicting the cleavage reaction of the wt substrate. Upon binding, relaxase bends its target in order to localize the *nic* site (N) within its active center. In presence of a divalent cation, the relaxase cleaves *nic* (blue arrowhead) and remains covalently bound to the 5’-phosphate of S, downstream from N. (D) Scheme depicting the cleavage reaction of the Rep-like substrate. The stem is bound by the relaxase, and the loop is located within the DNA binding cleft. Thus, the relaxase cleaves the scissile nucleotide within a stable cruciform (blue arrowhead). After cleavage the relaxase will be tethered to S and D. P is shown in yellow and U in purple. Blue triangles show the position of the *nic* site.

Relaxases and rolling-circle replicases belong to the HUH-endonuclease superfamily [1]. Replicases initiate replication of a large number of plasmids and viruses to generate new copies of their circular genomes [5]. Relaxases catalyze the transfer of one DNA strand of the plasmid genome to the recipient cell though a Type IV Secretion System during plasmid conjugation [6]. Both HUH endonucleases recognize their target ssDNA sites (called *nic* sites) with nanomolar specificity and form a protein-DNA covalent bond in presence of divalent cations. The linkage is a phosphotyrosyl intermediate between the catalytic tyrosine and the 5’ phosphate of the *nic* site. Moreover, they both carry out a second nucleophilic attack to the newly synthesized ssDNA strand, which results in recircularization of one unit of the ssDNA genome. Relaxases and replicases display structural similarity [1] but also outstanding differences. Structural data of relaxases and replicases revealed a similar fold (S1 Fig). In replicases, the HUH motif is located in a central five-stranded antiparallel β‑sheet [1]. The catalytic tyrosine is placed in an α-helix close to the C-terminal end of the replicase core. Relaxases can be understood as having suffered a circular permutation of the primary sequence with respect to replicases [1]. Thus, the catalytic tyrosine in relaxases is located at the N-terminus, while the HUH motif resides closer to the C-terminus (S1 Fig). Replicases extrude the dsDNA, allowing the formation of a cruciform structure and a stable *nic*-containing ssDNA loop [7,8]. On the other side, the accepted model of conjugation claims that the cruciform structure is not formed to attain the *nic*-cleavage reaction [9]. The relaxase binds the proximal arm of an inverted repeat (IR) and locates the *nic* site as ssDNA within the active center of the protein (Fig 1). Nevertheless, the IR forms a hairpin structure on ssDNA after conjugative replication (in the recipient cell), allowing the relaxase-catalized strand-transfer reaction that leads to circularization of the ssDNA. Most relaxases do not cleave *nic* efficiently unless relaxase-accessory proteins (RAPs) allow the generation of ssDNA. On the other hand, RAPs are not required for replicase activity. Thus, in order to facilitate the relaxase *nic*-cleavage reaction, we relocated the *nic* site within the loop of the IR, mimicking a replicase substrate. This substrate was further improved by exchanging the relative position of IR and *nic* sites. The results obtained provide a strategy for the design of more efficient substrates to be used *in vitro* for relaxase bionanotechnological reactions.

## Results

### TrwC_R_ cleaves substrates containing the *nic* site within the hairpin loop

Location of the *nic* site in their cognate substrate is structurally different in replicases, relaxases and other HUH endonucleases [1]. Despite the fact that HUH relaxases and replicases both recognize a hairpin-like structure, conjugative *nic* sites are located 3´ to the hairpin (Fig 1A) while replicase origins are located in the loop within the hairpin region (Fig 1B). The wt conformation recognized by relaxases possesses an inverted repeat (IR) that can be divided into a distal arm (D) and a proximal arm (P) with respect to the *n**ic* site (N). Moreover, there are eight nucleotides between P and N that are bent with a U-shape (U). The sequence (S) downstream N is tethered to the relaxase after cleavage (Fig 1C). In order to improve the relaxase *nic*-cleavage reaction, redesign of hairpin substrates was carried out by reconverting relaxase *nic* substrates into replicase like (Rep-like) substrates (Fig 1B). To create the Rep-like substrate, the 5´end of the distal sequence (D) was attached to the 3´end of the ssDNA region (S) after the *nic* site, thus creating a novel loop region (U+S) containing the *nic* site (N) and a dsDNA stem of 6 bp. The length of the loop (U+S) was tuned by including different number of nucleotides in the S region before the proximal arm (P) (see Table 1). Oligonucleotides with redesigned secondary structures were used to analyze *in vitro* the activity of the N-terminal relaxase domain of TrwC (residues 1 to 293) called TrwC_R_ hereafter.

**Table 1:** Cleavage activity of TrwC_R_ on different oligonucleotides.

*Nicking substrate*^a^	*DNA sequence*^b^	*Complex Formation %*^c^
R388 W(25+18)	GCGCACCGAAAGGTGCGTATTGTCT/ATAGCCCAGATTTAAGGA	**24.7** ± 2.1
R388 W(14+14)	GGTGCGTATTGTCT/ATAGCCCAGATTTA	**19.8** ± 1.9
R388 H(14+8)	GGTGCGTATTGTCT/ATCGCACC	**22.4** ± 5.7
R388 H(14+10)	GGTGCGTATTGTCT/ATAGCGCACC	**23.0** ± 4.7
R388 H(14+12)	GGTGCGTATTGTCT/ATAGCGCGCACC	**25.5** ± 3.1
R388 H(14+13)	GGTGCGTATTGTCT/ATAGCCGCGCACC	**23.3** ± 2.9
R388 H(14+14)	GGTGCGTATTGTCT/ATAGCCCGCGCACC	**26.8** ± 4.7
R388 H(14+15)	GGTGCGTATTGTCT/ATAGCCCAGCGCACC	**19.6** ± 1.4
R388 H(14+17)	GGTGCGTATTGTCT/ATAGCCCAGATCGCACC	**22.3** ± 6.1
R388 H(16+16)	CCGGTGCGTATTGTCT/ATAGCCCGCGCACCGG	**-**
R388 H(23+23)	AACCGGCTAGGTGCGTATTGTCT/ATAGCCCACGCACCTAGCCGGTT	**11,2**
R388 H(23+26)	AACCGGCTAGGTGCGTATTGTCT/ATAGCCCAGATCGCACCTAGCCGGTT	**13,5**
R388 H(24+24)	CCCAATGCGCGGTGCGTATTGTCT/ATAGCCCACGCACCGCGCATTGGG	**12,4**
R388 H(24+27)	CCCAATGCGCGGTGCGTATTGTCT/ATAGCCCAGATCGCACCGCGCATTGGG	**12,0**
R388H(24+31)	CCCAATGCGCGGTGCGTATTGTCT/ATAGCCCAGATCCACCGCACCGCGCATTGGG	**-**
R388 R(8+14)	TATTGTCT/ATAGCCCACGCACC	**30.2** ± 4.9
R388 R(0+27)	ATAGCCCAGATCGCACCGAAAGGTGCG	**5.8** ± 1.9
R388 R(1+27)	T/ATAGCCCAGATCGCACCGAAAGGTGCG	**9.9** ± 3.2
R388 R(4+27)	GTCT/ATAGCCCAGATCGCACCGAAAGGTGCG	**9.9** ± 3.9
R388 R(7+27)	ATTGTCT/ATAGCCCAGATCGCACCGAAAGGTGCG	**18.3** ± 11.6
R388 R(8+24)	TATTGTCT/ATAGCCCACGCACCGAAAGGTGCG	**45.3** ± 6.6
R388 R(8+27)	TATTGTCT/ATAGCCCAGATCGCACCGAAAGGTGCG	**44.7** ± 1.0
Rsf1010 WQ(30+7)	CAGTTTCTCGAAGAGAAACCGGTAAGTGCG/CCCTCCC	**43.9** ± 1.4
Rsf1010 WQ(23+7)	CCGGTTGAAAACCGGTAAGTGCG/CCCTCCCC	**41.4** ± 1.3
Rsf1010 HQ(18+16)	GAGAAACCGGTAAGTGCG/CCCTCCCCAGTTTCTC	**13.1** ± 4.2
Rsf1010 HQ(18+19)	GAGAAACCGGTAAGTGCG/CCCTCCCGATCAGTTTCTC	**9.0** ± 3.1
Rsf1010 HQ(18+22)	GAGAAACCGGTAAGTGCG/CCCTCCCTAGCCCCAGTTTCTC	**27.8** ± 4.5
Rsf1010 RQ(8+28)	TAAGTGCG/CCCTCCCCAGTTTCTCGAAGAGAAACCG	**-**
Rsf1010 RQ(8+34)	TAAGTGCG/CCCTCCCAGCCCCCAGTTTCTCGAAGAGAAACCG	**1.4**
Rsf1010 RQ(8+40)	TAAGTGCG/CCCTCCCAGCTGAATGTTCGAGTTTCTCGAAGAGAAACCG	**-**
RP4 WP(24+8)	GTGAAGGAAACTTCACCTATCCTG/CCCGGCTG	**21.3** ± 0.9
RP4 WP(15+6)	ACTTCACCTATCCTG/CCCGGC	**24.4** ± 5.7
RP4 HP(14+14)	CTTCACCTATCCTG / CCCGGCTGGTGAAG	**-**
RP4 HP(14+21)	CTTCACCTATCCTG / CCCGGCTGTACCTACGTGAAG	**-**
RP4 RP(8+24)	CTATCCTG/CCCGGCTGGTGAAGGAAACTTCAC	**23.7** ± 2.7

^a^ Nicking substrate names contain the plasmid name followed by either W (wild type) H (hairpin) or R (reverse), family group (Q for MOB_Q_ and P for MOB_P_, omitted in R388 MOB_F_) and the sequence length. + represents the *nic* site, and the number after it, the oligonucleotide length which remains bound to the relaxase after cleavage.

^b^ The slash in the DNA sequence depicts the *nic* site. The underlined sequences highlight the inverted repeats.

^c^ The cleavage activity of the relaxase was measured by the formation of relaxase-DNA covalent complexes in SDS-PAGE. Values are the average of three experiments, but R388 H(23+23),H(23+26), H(24+24), H(24+27) and RSF1010 RQ(8+34) that were not replicated.

**Fig 2:**
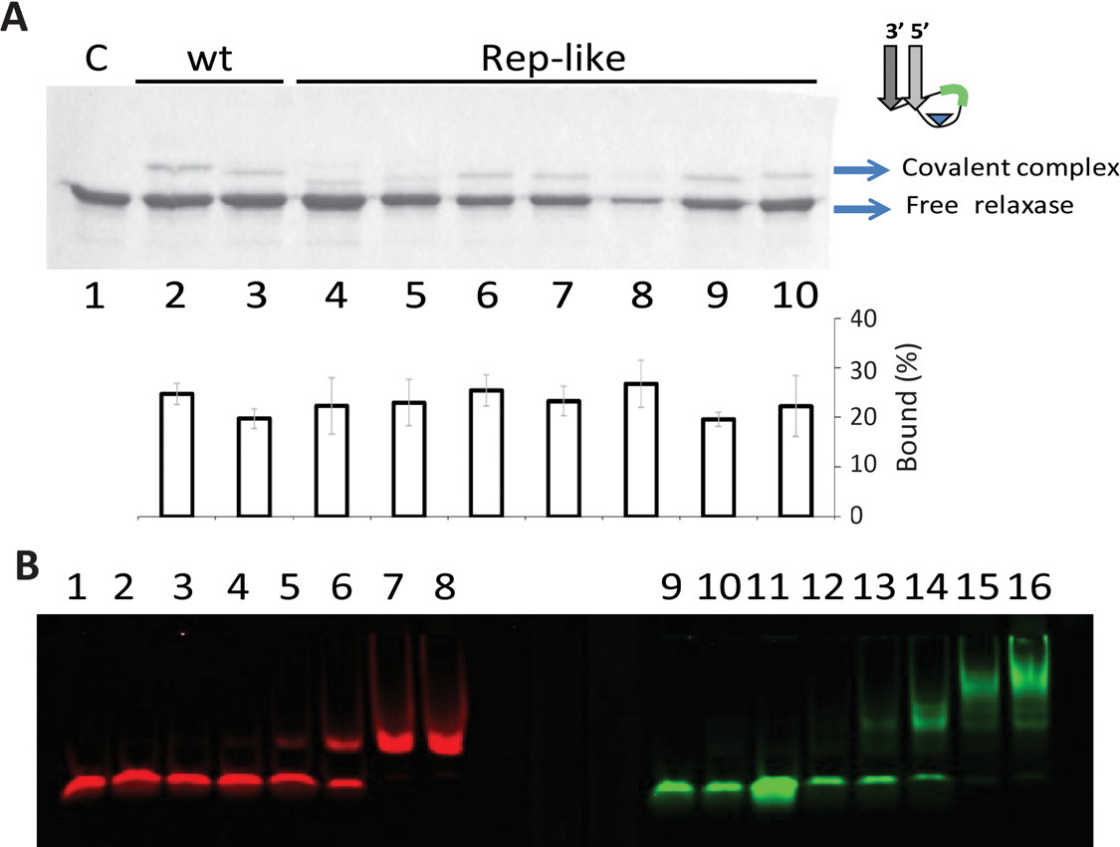
Interaction of TrwC_R_ with Rep-like substrates. (A) SDS-PAGE of oligonucleotides with R388wt or Rep-Like structures, when incubated with TrwC_R_. 6 μM TrwC_R_ was incubated with 15 μM of different oligonucleotides. The reaction products were separated by electrophoresis in 12% SDS-PAGE gels. Lane 1, no oligonucleotide; Lanes 2 and 3, R388wt oligonucleotides W(25+18) and W(14+14), respectively; in subsequent lanes, TrwC_R_ was incubated with Rep-like oligonucleotides. The S length of Rep-like substrates (in green), varies from two to eleven nucleotides. Lane 4, H(14+8), Lane 5, H(14+10), Lane 6, H(14+12); Lane 7, H(14+13), Lane 8, H(14+14); Lane 9, H(14+15); Lane 10, H(14+17). In the center chart, percentage of bound complexes were calculated in three separate experiments such as that shown in (A). (B) Increasing amounts of TrwC_R_ were incubated with wt oligonucleotide W(25+8) (red shift, lanes 1 to 8) or Rep-like hairpin H(14+14) (green shift, lanes 9–16). Lanes 1 and 9, no protein added; 2 and 10, 42 nM of TrwC_R_; 3 and 11, 85 nM; 4 and 12, 210 nM; 5 and 13, 420 nM; 6 and 14,850 nM; 7 and 15, 4,2 μM, 8 and 16, 8,5 μM.

The effect of the DNA substrate length and secondary structure on relaxase cleavage was investigated through *in vitro nic*-cleavage reactions. This reaction generates a protein-DNA covalent complex that can be quantified by its lower mobility using SDS-PAGE as described in Materials and Methods. TrwC_R_ was able to cleave and remain covalently bound to oligonucleotides containing IR_2_-*nic* (W(25+18)) or P-*nic* (W(14+14)), (Fig 2A, Lanes 2 and 3). As previously described, the entire IR_2_ increases the binding affinity of TrwC_R_ but the distal arm is not required for efficient cleavage (9). Interestingly, TrwC_R_ could also cleave a Rep-like oligonucleotide containing a loop with only 2 nucleotides in the S region (H(14+8)) (Fig 2, lane 4). TrwC_R_ cleavage activity on H(14+8) was similar to that on W oligonucleotides. Incubation of TrwC_R_ with Rep-like hairpins containing longer loops, such as H(14+10), H(14+12) or H(14+13), resulted in a band with reduced mobility, as expected by the formation of a TrwC_R_ covalent complex with a decamer (Lane 5), dodecamer (Lane 6) and tridecamer (Lane 7), respectively. Similar results were obtained with substrates H(14+14), H(14+15) or H(14+17) (Lanes 8, 9 and 10, respectively). Labeled Rep-like oligonucleotide H14+14 was also found to be effectively cleaved by TrwC_R_ using TBE-urea gel electrophoresis analysis (S2 Fig).

TrwC_R_ protein was previously shown to cleave oligonucleotides containing the R388 *nic* site and to perform an *in vitro* strand transfer reaction to a second oligonucleotide also containing the *nic* site [10,11]. Since TrwC_R_ cleaves Rep-like oligonucleotides and remains covalently attached to the oligomer downstream *nic* (Fig 1D), it could carry out the strand transfer reaction. When a labeled wt oligonucleotide acceptor F(25+0) was provided and incubated with TrwC_R_ and oligonucleotide H(14+8), 29% of the labeled oligonucleotide shifted to a position corresponding to a F(25+8) oligonucleotide (Table 2 and S3 Fig). Compared to the transfer reaction using wt oligonucleotides as donors, this transfer efficiency is similar to W(14+14) (31%) and lower than W(25+18) (41%). Longer Rep-like oligonucleotides, such as H(14+10) and H(14+14), generated around 17% product. Overall, all Rep-like substrates were efficiently transferred to receptor oligonucleotides (see Table 2).

**Table 2:** Strand transfer reaction catalyzed by TrwC_R_ of oligonucleotides with the Rep-like and Reverse-like layout to the W(25+0) oligonucleotide.

*Nicking substrate*^a^	*Transfer activity %*^b^
R388 H(14+8)	**29** ± 8
R388 H(14+10)	**17** ± 5
R388 H(14+12)	**19** ± 7
R388 H(14+13)	**20** ± 10
R388 H(14+14)	**17** ± 2
R388 H(14+15)	**13** ± 10
R388 H(14+17)	**10** ± 6
R388 R(8+14)	**2** ± 3
R388 R(7+27)	**3** ± 3
R388 R(8+24)	**2** ± 2
R388 R(8+27)	**4** ± 2
R388 W(14+14)	**31** ± 2
R388 W(25+18)	**43** ± 1

^a^ Substrates are named as in Table 1.

^b^ The strand transfer activity of the relaxase was measured by the formation of fluorescent W(25+x) oligonucleotides in denaturing PAGE. Values are the average of three experiments.

We thought that the distance (S) from *nic* (N) to the proximal arm (P) would be critical for cleavage, since only certain loop lengths would allow the *nic* site to reach the active site of the protein. As similar *nic*-cleavage efficiencies were observed regardless of loop length (Fig 2A), gel shift assays were performed to study how TrwC_R_ interacts with Rep-like conformation substrates. Increasing concentrations of TrwC_R_ were incubated with 50 nM of a green-fluorescent labeled Rep-like oligonucleotide H(14+14) or with a red-fluorescent labeled wt oligonucleotide W(25+8). Although the EC_50_ (half maximal effective concentration) was the same with both oligonucleotides, only one shifted band was observed with W(25+8), while several shifted bands appeared with H(14+14). Both unbound oligonucleotides produced a single band (Fig 2B, lane 1 and 9, respectively). Thus, upon TrwC_R_ binding, W(25+8) seems to form a unique complex, while different complexes are formed with H(14+14).

The interaction profile of Rep-like oligonucleotides incubated with TrwC_R_ was further analyzed by gel filtration. Consistent with the EMSA result, several peaks with higher molecular weight than the predicted peak for 1:1 complexes appeared when either Rep-like H(14+14) and H(16+16) (S = 8), H(14+15) (S = 9) or H(14+17) (S = 11) oligonucleotides were incubated with TrwC_R_ (S4 and S5 Figs). It is likely that TrwC_R_ could melt the hairpin and stabilize a “linear” DNA in a way that the distal and proximal arms of the IRs of different DNA molecules could interact, creating these higher molecular weight complexes.

In order to stabilize the stem-loop structure of Rep-like substrates to avoid the formation of intermolecular complexes, longer IRs were used (H(23+23), H(23+26), H(24+24), H(24+27), H(24+31)). Now, a sole band of protein-DNA was shown in chromatography, while SDS-PAGE gels revealed that the covalent complexes are still being formed with longer stem substrates (S6 Fig).

### Rep-like substrates are cleaved by single-Y relaxases

**Fig 3:**
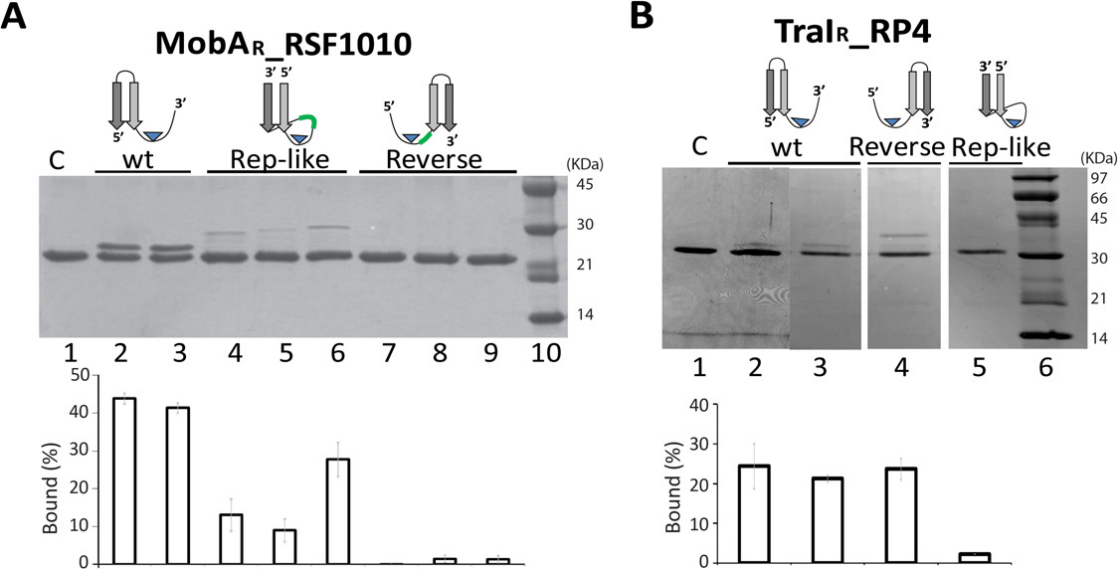
Novel designs for DNA substrates of model single-Y relaxases. (A) SDS-PAGE of MobA_R__RSF1010 with its targets. 7 μM MobA_R_ was incubated with 15 μM of different oligonucleotides. Lane 1, MobA_R_. Lanes 2 and 3, wt oligonucleotide WQ(30+7) and a substrate that lacks the upper-hairpin nucleotides of the IR WQ(23+7). Lanes 4, 5 and 6, Rep-like oligonucleotides HQ(16+16),HQ(16+19) and HQ(16+22), respectively. Lanes 7, 8 and 9 reverse substrates RQ(8+28), RQ(8+34) and RQ(8+40). Lane 10, molecular weight ladder. (B) SDS-PAGE of TraI_R__RP4 with its targets. 1.5 μM TraI_R_ was incubated with 15 μM of different oligonucleotides. Lane 1, TraI_R_; lane 2, wt substrate WP(15+6); lane 3, wt substrate WP(24+8); lane 4, reverse substrate RP(8+24) and lane 5, Rep-like substrate HP(14+14). Molecular weight ladder is shown on Lane 6. Bar graphs with the quantification of covalent complexes are shown below the SDS-PAGE gels. Data showed mean±s.d. of three independent experiments.

Relaxases contain either one (Y1) or two (Y2) catalytic tyrosines [1,12]. The ssDNA U-turn observed in Y2 relaxase domains TrwC_R_- and TraI_R_ -DNA complex structures was also observed in the structure of Y1 relaxase NES [13–15]. However, relevant differences in U-turn formation have been described [12]. In order to check if Y1 relaxases could also catalyze cleavage of Rep-like substrates, we analyzed these substrates for the best known Y1-relaxases, MobA_RSF1010 and TraI_RP4 (S7 Fig). The MobA_RSF1010 binding site was identified as a 10 bp IR that forms a hairpin structure upstream from *nic* [16]. Mozingo et al. modeled the relaxase minMobA (amino acids 1 to 186, named MobA_R_ in this study) complexed with a 33-mer oligonucleotide and compared this model with TrwC_R_ bound to the 23-mer [16]. According to this comparison, only nucleotides at the base of the 10 bp-hairpin interact with MobA_R_. To study the influence of IR length, we designed a shorter wt substrate, containing a 6 bp IR WQ(23+7) (Fig 3A and Table 2). Analysis by SDS-PAGE of the covalent complexes obtained after incubation with the protein showed that either the long wt substrate WQ(30+7) (Fig 3A Lane 2) or the short wt substrate WQ(23+7) (Fig 3A Lane 3) generated 40% covalent complexes. These results confirm that just the nucleotides at the base of the stem make specific interactions with relaxase MobA_R_.

Rep-like substrates of RSF1010 *nic-site* sequence were designed as for R388 *nic* (Fig 1). Three different loop lengths (S) were tested, while maintaining the first 18 nucleotides invariant (P-U-N). The three different oligonucleotides tested, HQ(18+16), HQ(18+19) or HQ(18+21), showed reduced activity compared with wt Q oligonucleotides. MobA_R_ cleaved the Rep-like substrate HQ(18+16) to 13% (Fig 3A Lane 4). HQ(18+19) (Lane 5) behaved similarly, producing 10% covalent complexes. In turn, HQ(18+21) (Lane 6) generated 20% covalent complexes (Table 2). These results suggest that Rep-like substrates also allow the correct location of the *nic* site in the catalytic center of the relaxase MobA_R_.

Another widely characterized Y1 relaxase is TraI_RP4 [17–19]. The DNA substrate routinely used in TraI cleavage assays was the 21-mer oligonucleotide WP(15+6). None of the previous assays had used any substrate carrying an IR upstream from *nic*. In our study, a substrate with a 6-bp hairpin conformation WP(24+8) was designed. As shown in Fig 3B, lane 3 WP(24+8) was efficiently cleaved by a protein containing the 270 N-terminal residues of RP4 TraI (TraI_R_). Covalent complex formation by the wt substrate was slightly lower than when WP(15+6) was used (Lane 2 and Table 2).

We then used a rational approach to design DNA stem-loops that could be cleaved by TraI_R_. Rep-like oligonucleotides containing the minimal RP4 *nic* sequence were developed as we did before for R388 (P-U-N-S-D, see Fig 1). These substrates were incubated with TraI_R_, and the resulting complexes where analyzed by SDS-PAGE. Neither Rep-like oligonucleotide HP(14+14) (Fig 3B Lane 5) nor HP(14+21) were cleaved, even though saturating concentrations of oligonucleotides where used (data not shown).

### Improved scissile substrates were obtained by permutating the *nic* sequence

**Fig 4:**
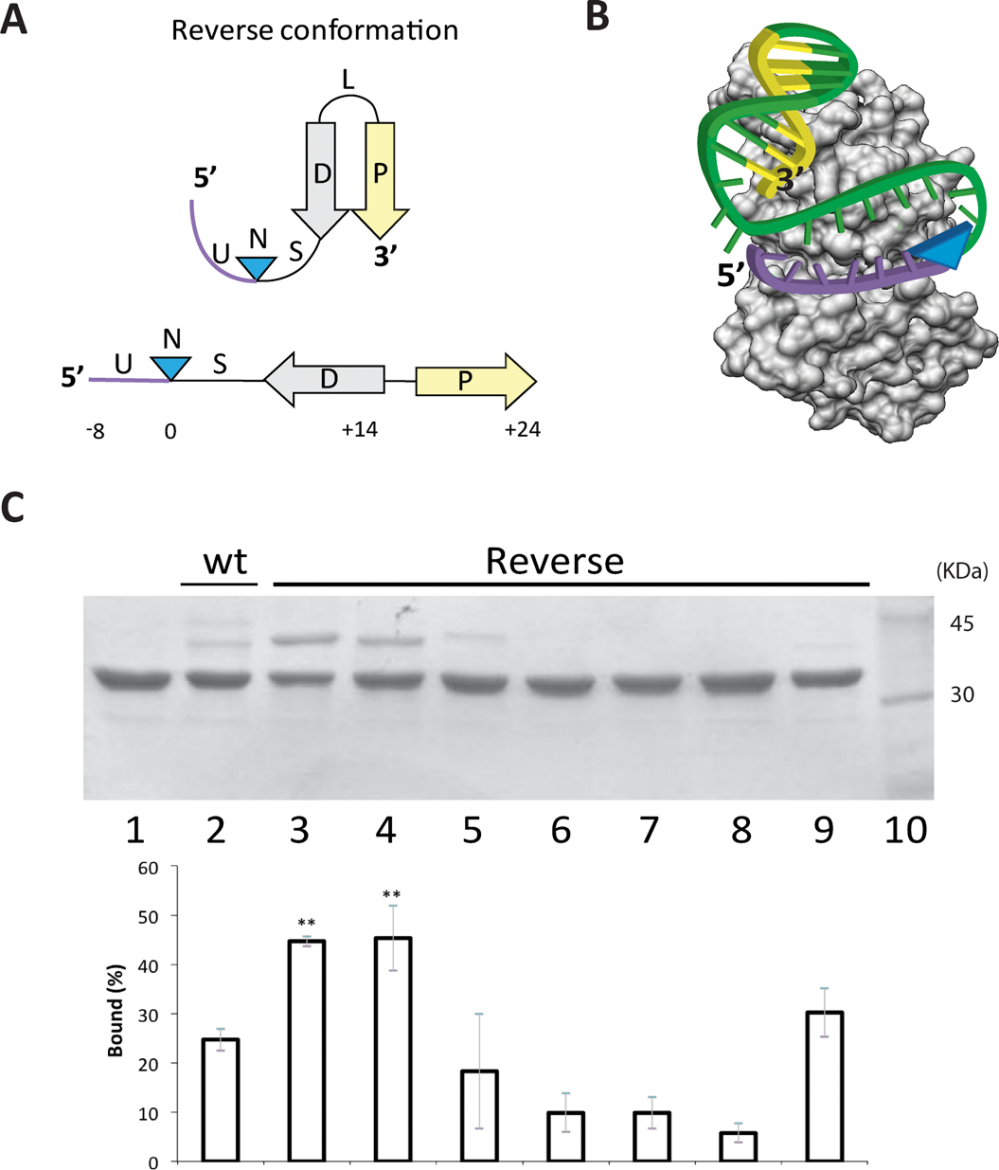
Interaction of TrwC_R_ with Reverse substrates. (A) Reverse substrates were designed by swapping the 5’ region of the *nic* site to the 3’ end. This designed DNA substrate possesses the complete inverted repeat (D-P) at the 3’ end of the *nic* site (N). Either the U or the S lengths were tuned to allow the correct location of the hairpin within the relaxase binding domain. (B) Scheme depicting the cleavage reaction of the reverse substrate. Relaxase binding to the reverse substrate allows both the hairpin and the single strand U-turn localize at the DNA binding cleft. This way the cleavage reaction forms a covalent complex of the relaxase with the region downstream of the *nic* site (blue arrowhead). Now the 5´side of *nic* do not contain the IR avoiding the re-ligation reaction. Color code is the same than Fig 1. (C) 12% SDS-PAGE of reverse oligonucleotides, when incubated with TrwC_R_. 6 μM TrwC_R_ was incubated with 15 μM of different reverse oligonucleotides. Lane 1, no oligonucleotide; Lanes 2, R388wt oligonucleotide W(25+18). Lanes 3 and 4, Reverse substrates R(8+27) and R(8+24), both with U = 8 nt and S = 11nt or S = 8 nt respectively. Lane 5, R(7+27) U = 7; Lane 6, R(4+27) U = 4; Lane 7, R(1+27) U = 1 and Lane 8, R(0+27) U = 0. Lane 9, R(8+14), U = 8 P = 0. Lane 10, Molecular weight marker. Graph quantifying the percentages of covalent complexes is shown below the SDS-PAGE gel. Data show mean±s.d. of three independent experiments. Two asterisks indicate P-value<0.05 by two-sided student’s t-text.

In TrwC_R_ wt substrate W(25+8), the stem loop is non-covalently bound by the relaxase after cleavage, while the 8-mer downstream *nic* is covalently attached to the catalytic tyrosine. There is a cleavage-ligation equilibrium with wt oligonucleotides, because the 25-mer oligonucleotide remains in the protein DNA binding domain after cleavage and, therefore, the 8-mer can be easily religated (Fig 1C). We thought that, by linking the 3´ end of *nic* (U-N-S) to the 5´end of the hairpin, the 5´side of *nic* (the single strand U sequence upstream the *nic* site) would lose binding affinity (Fig 4A). As a consequence, the resulting 5´ssDNA could be released from the relaxase, avoiding religation and displacing the reaction equilibrium towards the formation of covalent complexes (Fig 4B). Under this rationale, novel substrates were designed by shuffling the wt sequence in the order U-N-S-D-P. These synthetic oligonucleotides were called “reverse substrates” because the hairpin is located downstream from the *nic* site.

R388 reverse oligonucleotides were designed using either the whole U ssDNA (8 nt before *nic*) or a fragment (up to seven nt), which would be liberated after cleavage. Besides, S length varied from 8 to 11 nucleotides, to allow the correct disposition of the hairpin into the relaxase. As shown in the SDS-PAGE gel of Fig 4C, the relaxase band shifted when incubated with R(8+24), R(7+27) or R(8+27) reverse oligonucleotides. In fact, the percentage of protein-bound DNA boosted with reverse oligonucleotides R(8+27) (Lane 3) and R(8+24) (Lane 4) to almost 50% when U had 8 nt. It is noteworthy that the covalent complex decreased to 18% when the U length was 7 nt (lane 5). Moreover, the covalent product was not detected when the U-region was shorter, such as when oligonucleotides R(4+27) (Lane 6), R(1+27) (Lane 7) or R(0+27) (Lane 8) were used. The different yields of covalent complexes revealed that U length is a determinant factor for the reaction, while S length had no significant influence.

Regarding Y1 relaxases, reverse substrate RP(8+24) was recognized and cleaved by TraI_R_ _RP4 (Fig 3B Lane 4). The amount of covalent complex obtained with RP(8+24) was comparable to the amount obtained with wt WP(24+8) oligonucleotide (Table 1). Interestingly, MobA_R_ could not cleave similarly designed oligonucleotides RQ(8+28), RQ(8+34) or RQ(8+40) (Fig 3A Lane 7, 8 and 9, respectively).

### Plasmids carrying novel *nic*-site conformations show diminished conjugation rates

**Fig 5:**
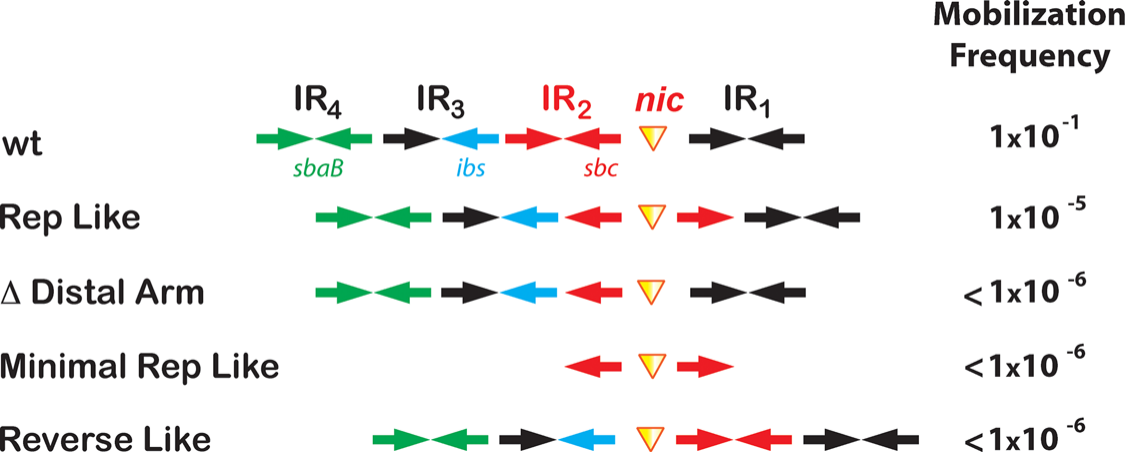
Mobilization frequencies of synthetic *ori*T-plasmids. Mobilization frequencies obtained using pSU2007 as helper plasmid are expressed as number of transconjugants per donor cell. Values are the average of three experiments. The relative position of the secondary structure elements of the assayed synthetic *oriT*s is shown. R388 wt *oriT* has four IR that are binding sites for the relaxase and nicking-accessory proteins TrwA and IHF. TrwC binds IR_2_ and the *nic* site (*sbc*, red), TrwA binds IR_4_ (*sbaB*, green) while IHF binds IR_3_ (*ibs*, blue). The distance between secondary structure elements is not shown at scale.

The stable interaction of TrwC_R_ with the novel substrates supports the notion that both Rep-like and reverse oligonucleotides could modify the *in vivo* efficiency of relaxases. The importance of the DNA stem loop structure for efficient relaxase-mediated DNA processing *in vivo* was investigated by using plasmids containing synthetic *oriT*s. Synthetic R388 *oriT*s were designed by substitution of the wt *nic*-cleavage site (IR_2_+*nic*) by either H(14+14) Rep-like or R(8+24) Reverse-like sequences (Fig 5 and S8 Fig).

*OriT*-containing plasmids (Cm^R^) can be mobilized to a recipient cell (strain UB1637; Sm^R^) in the presence of the helper plasmid pSU2007 (Km^R^). Mobilization frequencies of the synthetic *oriT*-containing plasmids were measured as the number of mobilized plasmids (Cm^R^Sm^R^) per donor cell (Cm^R^Nx^R^) (Fig 5). The Rep-like *oriT* plasmid showed 10^4^ fold lower mobilization than the wt o*riT* plasmid. By substitution of the Rep-like site for the wt *nic*-site, not only the relaxase recognition site was changed, but also the distance between the TrwC and TrwA/IHF binding sites. TrwA binding to *oriT* was shown to be essential for proper conjugation [20]. In the Rep-like *nic*-site, TrwC and TrwA binding sites were 10 nucleotides closer. Thus, we also analyzed the mobilization frequency of a synthetic plasmid that lacks the distal arm (ΔDistal Arm *oriT*). Interestingly, the conjugation rate of ΔDistal Arm *oriT* plasmid dramatically dropped below detection levels (<10^−6^ transconjugants/donor).

We also checked the conjugation ability of a plasmid containing just the Rep-like *nic* site without any binding sites for IHF and TrwA, named minimal Rep-like. Mating carried out with minimal Rep-like *oriT* did not generate transconjugants. Similarly, reverse-like plasmids, which have the *nic* site placed upstream IR_2_, also resulted in a substantial reduction of plasmid conjugation.

## Discussion

In this work, we analyzed if relaxases would recognize and cleave substrates with a replicase *nic* site layout. Thus, we designed Rep-like substrates with secondary structures mimicking the stem-loop recognized and cleaved by rolling circle replicases (Fig 1B). We observed that TrwC_R_, the relaxase of plasmid R388, cleaved Rep-like *nic*- sites with a yield similar to wt substrates. Similar Rep-like substrates were designed and assayed for Y1 relaxases MobA_R__RSF1010 and TraI_R__RP4. MobA_R_ efficiently cleaved the corresponding Rep-like substrates (Fig 3A) without melting the DNA to cleave the *nic* site. However, despite having found an effective wt hairpin substrate that is cleaved by TraI_R__RP4, this relaxase did not cleave Rep-like oligonucleotides (Fig 3B).

Although Rep proteins effectively cleave oligonucleotides mimicking their cognate substrates *in vitro* [21–23], in general relaxase activity with Rep-like substrates was lower than with their cognate substrates. The most likely explanation is that in Rep-like substrates the D and P segments of the IR are located downstream and upstream from N, respectively. Thus, now the relaxase tightly binds both sides of the *nic* site favoring the religation process (Fig 1D). To avoid this situation, we designed novel reverse substrates where the entire IR is now located downstream from N (Fig 4B). Using this substrate, relaxase contacts 5´ of *nic* are reduced and the religation reaction is diminished. Even though the balance was displaced to DNA-protein complex formation, 100% yield could not be achieved, probably because the relaxase has residual affinity for the U-turn 5´side of the cleaved reverse oligonucleotide. TraI_R_ also cleaved the reverse substrate RP(8+24) although, unexpectectly, the yield of covalent complex formation with the novel substrates did not improve (Fig 3B). On the other hand, MobA_R_ did not cleave reverse-oligonucleotides. This protein lacks the fingers domain at the C-terminal end. The fingers domain shapes a cleft that stabilizes the U-turn in the active center in MOB_F_ relaxases (S1 Fig). Probably this feature allows MobA_R_ to form 40% covalent complexes with wt oligonucleotides WQ(30+7) and WQ(23+7) (Fig 3A), because religation is limited. We hypothesize that this feature could restrict the cleavage of reverse substrates. Overall, the cleavage-ligation reaction is tightly in balance for each relaxase, and not all relaxases can improve the yield of covalent complexes.

Once demonstrated that *in vitro* reactions were enhanced by novel relaxase substrates, we analyzed their behavior in whole cell experiments. With the goal of improving the efficiency of relaxases within living cells, synthetic plasmids in which the relaxase target was converted to either Rep-like sequence H(14+14) or Reverse R(8+24) were tested. Conjugation rates critically fell for all synthetic plasmids tested (Fig 5). Rep proteins can cleave dsDNA plasmids in the absence of RAPs because the *nic* site is at the loop of a cruciform IR [24]. However, most relaxases need RAPs to locally alter DNA supercoiling in a way the relaxase can melt the DNA to display the *nic*-containg ssDNA [25–27]. In the conjugation assay of Rep-like and reverse *nic* containing plasmids, we changed the relative orientation of *nic* with respect to TrwA (*sbaB*) and IHF (*ibs*) binding sites. Moreover, ssDNA hairpin formation by the modified IR region is different in recipient cells and could affect the termination reaction. It has been described for relaxases as well as replicases that the sequence requirements for the termination are different than the requirements for initiation [7,9]. Further studies will focus on the directed evolution of these synthetic *ori*Ts to obtain transferable plasmids in the absence of RAPs.

The significance of the *in vitro* results derives from the finding of new cleavable substrates for HUH proteins by rational design. Results show that relaxase TrwC_R_ acting on reverse *nic* sites is more efficient in cleavage than when acting on its cognate sites. Relaxases are bifunctional proteins, having an N-terminal relaxase domain composed of roughly 300 residues fused to a C-terminal domain with various biochemical activities (helicase, primase, etc). The relaxase domain can be fused with heterologous proteins (e.g., fluorescent proteins) without losing activity. Since relaxases (and relaxase proteins fused to fluorescent proteins) attach covalently to a specific sequence of DNA, even on DNA nanostructures [3], our results are promising for nanobiotechnology applications using DNA nanostructures, where efficient methods for bioconjugation are needed. In an ideal case, the ability of relaxases to form covalent bonds with DNA nanostructures could be used for creating nanofactories. The relaxase domains would act as linkers between catalytic proteins (e.g., proteins involved in a given biochemical pathway) and the DNA nanostructures [28]. Relaxases can be tethered to specific locations in 2D and 3D DNA nanostructures so that any protein of interest can be placed in a precise and programmable mode [29]. As a second set of applications, TrwC is a potential tool for human genome editing [30]. By widening the number of sequences that TrwC and other relaxases can recognize, the potential targets for genome integration and targeted modification of the human genome could be greatly increased [4]. The present results have to be considered together with previous data where crucial nucleotides involved in target selection were identified [12]. Here we show that these targets could be expanded by the use of permuted sites. Overall, our results put relaxases among the most versatile tools for bioconjugation to DNA nanostructures.

## Materials and Methods

### Protein purification

The N-terminal relaxase domain of TrwC (TrwC_R_, amino acid residues 1 to 293) was purified as previously described [14] with minor modifications. TrwC_R_ expression from plasmid pSU1588 (pET3a:*trwc*_*R*_) in *Escherichia coli* strain C41 was induced with 0.5mM IPTG for 3 h. Cells were harvested by centrifugation and stored at -80°C. Frozen cells were thawed at 37°C, resuspended in a solution containing Tris 100 mM (pH = 7.6), NaCl 500 mM, EDTA 1mM, PMSF 1% and lysed by 10 min. sonication in pulses. The lysate was centrifuged at 40,000g for 15 min at 4°C. Supernatants were applied to a P11-phosphocellulose column equilibrated in buffer A (50 mM Tris–HCl pH 7.6, 0.1 mM EDTA) containing 200 mM NaCl and eluted with buffer A with 600 mM NaCl. Fractions containing TrwC_R_ were pooled, diluted to 200 mM NaCl, loaded onto a Hi-Trap SP HP column (GE Healthcare), and eluted with a linear NaCl gradient (200 mM–1000 mM NaCl) in buffer A.

The N terminal fragment of RSF1010 *mobA*, which codes for the first 186 residues of MobA (called minMobA by Mozigo et al.[16] and here named MobA_R_), was cloned in pET3a expression vector between *Nde*I and *Bam*HI restricition sites (pSU10064). Expression of MobA_R_ was achieved using *E*.*coli* C41 strain. After induction for 3 h with IPTG, cells were harvested and stored at -80°C. Purification was performed according to the procedure described for TrwC_R_.

The N terminal domain encoding the 270 residues of RP4 TraI (TraI_R_) was PCR amplified from plasmid RP4 and subcloned in pET3a using *Nde*I and *Bam*HI restriction sites, resulting in plasmid pSSP10. C41 strain carrying pSSP10 was induced for 3 h with IPTG. Cells were collected by centrifugation and stored at -80°C. RP4 TraI_R_ was purified as described above for TrwC_R_, except the Hi-Trap SP HP column was substituted by a Hi-Trap Heparin column, from which TraI_R_ was eluted with a linear 200–1000 mM NaCl gradient.

Gel filtration of all relaxases was carried out in a Superdex75 column 10/300 GL (GE Healthcare) equilibrated in 100 mM Tris–HCl (pH 7.6), 500 mM NaCl, 0.1 mM EDTA. All three relaxases eluted as a monomers of about 30 kDa for TrwC_R_, 30 kDa for TraI_R_ and 25 kDa for MobA_R_ (see S7 Fig). Protein concentration was estimated by UV absorbance at 280 nm in a Nanodrop spectrophotometer, using extinction coefficients of 35410 M^-1^ cm^-^1 (Abs 0.1% of 1.078) for TrwC_R_, 32430 M^-1^ cm^-1^ (Abs 0.1% of 1.551) for MobA_R_ and 20190 M^-1^ cm^-1^ (Abs 0.1% of 0.636) for TraI_R_. After Superdex75 column chromatography, relaxase containing fractions were pooled and stored at -80C for further use.

### ssDNA- Protein complex formation

Unlabeled DNA oligonucleotides were obtained from Sigma-Aldrich (St. Louis, MO). Their sequences are listed in Table 1. Oligonucleotides were resuspended in miliQ water at 100 μM, heated to 95°C for 10 min, and then either allowed to cool passively to room temperature or snap cooled on ice.

Cleavage reactions were carried out by incubating 6.3 μM TrwC_R_ with 15 μM of each oligonucleotide at 37°C for 1 h in 10mM Tris-HCl, pH7.6, 5mM MgCl_2_, 375 mM NaCl and 15 μM EDTA [11]. Similarly, MobA_R_ (7 μM) was incubated with 15 μM of each substrate in 25 mM Tris-HCl pH 8.0, 5 mM MgCl_2_, 225 mM NaCl and 15 μM EDTA [31]. For TraI_R_, the optimized reaction mixture was 10 mM Tris pH 7.6, 5 mM MgCl_2_, 225mM NaCl and 15 μM EDTA [17]. 1.5 μM of TraI_R_ was incubated with 15 μM oligonucleotide in each reaction. Reactions were stopped by adding SDS and boiling the samples. The cleavage activity was checked by the lower mobility of the protein-DNA covalent complexes in SDS-PAGE. There was always a 2:1 molar excess of oligonucleotides to guarantee that all the protein is in complex with the oligonucleotide. DNA nicking activity was expressed as percentage of covalent complexes generated (the intensity of the product band divided by the sum of product and free protein band intensities). Each data point represents the average of three reactions. Data were processed using Quantity One (Biorad, Hercules, CA).

### IRDye-labelled oligonucleotide cleavage assays

Cleavage reaction mixtures (20 μL) contained 50 nM IRDye-labelled oligonucleotide W(25+8) or H(14+14), 1 μM TrwC_R_ in 10 mM Tris-HCl, pH7.5, 5 mM MgCl_2_, 100 mM NaCl. After incubation for 1 h at 37°C, reaction mixtures were digested with 0.6 mg/ml proteinase K and 0.05% (w/v) SDS for 20 min at 37°C. Reactions were run through a denaturing TBE-urea polyacrylamide gel at 200V for 70 min to separate cleaved product DNA from the substrate. Oligonucleotides were visualized using an Odyssey Infrared Image System (LI-COR Biosciences).

### Strand-transfer assays

For oligonucleotide strand-transfer reactions, mixtures contained 0.25 μM of Rep-like oligonucleotides (Table 1) and1 μM of protein TrwC_R_ in 10 mM Tris- HCl pH 7.6, 5 mM MgCl_2_, 110 mM NaCl and 15 μM EDTA. After 30 min, 50 nM IRDye800-labelled oligonucleotide R388 W(25+0) (5´- CGCACCGAAAGGTGCGTATTGTCT) was added to the reaction mixture. After incubation for 30 min at 37°C, reaction mixtures were digested with 0.6 mg/ml proteinase K and 0.05% (w/v) SDS for 20 min at 37°C. Oligonucleotide separation was performed by electrophoresis in a denaturing 18% Acrylamide, 8 M Urea gel at 200V for 70 min. Images were processed with Odyssey Infrared Image System (LI-COR Biosciences) and quantified by Quantity One software (Biorad).

### Electrophoretic Mobility Shift Assay (EMSA)

TrwC_R_ binding to IRDye800-labelled oligonucleotide R388 W(25+8) (CGCACCGAAAGGTGCGTATTGTCT/ATATTGTCT), or IRDye700-labelled R388 H(14+14) (GGTGCGTATTGTCT/ATAGCCCGCGCACC) was analyzed by EMSA. Binding reactions contained 50 nM IRDye-labeled oligonucleotide and increasing concentrations of TrwC_R_ in buffer (10 mM Tris-HCl pH 7.6, 100 mM NaCl, 1 mM EDTA). Reaction mixtures were incubated for 30 min at room temperature and loaded onto a 10% non-denaturing polyacrylamide gel. After electrophoresis at 200 V during 15 min, images were processed and quantified using Odyssey Infrared Image System (LI-COR Biosciences).

### Intra- and intermolecular structures of *nic*-site oligonucleotides

Analysis of intra- and intermolecular structures in the oligonucleotides library created for the determination of the best substrate for TrwC_R_ was determined by gel filtration in a Superdex75 PC 3.2/30 (GE Healthcare). 2.0 μl of each oligonucleotide (100 μM) were diluted in 18 μl of buffer solution (100 mM Tris pH 7.6, 200 mM NaCl, 10 mM EDTA) and injected in the column loop of 25 μl. The elution buffer was 100 mM Tris 7,6, 200 mM NaCl, 1 mM EDTA. When we analyzed the protein, 20 μl of stock solution (42 nM of TrwC_R_) was injected alone. The complexes of TrwC_R_ with oligonucleotides were loaded after 30 min incubation at room temperature, with an excess of oligonucleotide (ratio 1:1.5) to ensure maximum complex formation.

To study the new balance achieved with Rep-like substrates, IRDye700 labeled oligonucleotide R388 H(14+14) mixed with TrwC_R_ in presence of 10 mM Mg_2_^+^ during 1 h was analyzed by gel filtration and native electrophoresis. After high resolution gel filtration column chromatography of the binding mixture, 10 μl of the fractions were loaded into a 5% non-denaturing acrylamide gel, and the fluorescent label of the oligonucleotide was detected with Odyssey infrared scanner.

### Conjugation experiments

The Rep-like and Reverse-like *oriT* plasmids were design and ordered to GeneArt (Life technologies-Invitrogen). The ΔDistal Arm plasmid resulted from pSU1186 PCR-amplified with primers containing the deletion of the distal arm of IR_2_. The linearized plasmid was self-circularized with T4 ligase. For minimal Rep-like plasmid, two oligonucleotides with the 28 base pairs Rep-like sequence between *Bam*HI and *Hind*III sites were cloned in the pSU19 digested vector.

Matings were carried out by the plate-mating procedure described in [32]. DH5α (Nx^R^) cells containing the helper plasmid pSU2007 (a Km^R^ derivative of R388) [9,33] and the mobilizable synthetic *oriT*-containing plasmids to be checked (Cm^R^) were mated with strain UB1637 (Sm^R^) as a recipient strain. Saturated cultures of donor and recipient strains were mixed in a 1:1 ratio and mated on a LB agar surface. After 1 h at 37°C, serial dilutions were plated. Mobilization frequencies were expressed as number of Cm^R^ transconjugants (Cm^R^Sm^R^) per donor cell.

## Supporting Information

S1 Fig Comparison of the 3D structure of relaxases and replicases. A) Organization of the catalytic centre in Relaxases (TrwC_R_ from R388 plasmid bound to its target (PDB 1OMH)) and RCR replicases (AAV with RBE (PDB 1UUT)). Relaxases and RC-Rep suffered a circular permutation in the primary sequence that localizes the catalytic tyrosine at the N-terminal in the relaxases, but close to the C-terminal in Rep proteins. Relaxases recognize the *nic* site (red triangle) 5´ to an inverted repeat, while Reps cleave a *nic* site within a stem-loop. B) Ribbon structure of relaxases (left) and RC-Reps (right). TraI_R_ from plasmid pCU1 (PDB 3L57) and TraI_R_ from plasmid F (PDB 2AOI) belong to MOB_F_ family of relaxases whereas NES from pLW1043 (PDB 4HT4) and minMobA frm R1162 (PDB 2NS6) belong to MOB_Q_ family of relaxases. RepB, the RC initiators of plasmid pMV158 (PDB 3DKX) and Rep from geminivirus (PDB 1L5I) also have a similar folding. All these proteins possess a core of five antiparallel β-strand, where the HUH motif is located at the third β-strand, near the α-helix in which the catalytic tyrosine(s) is held.
(PDF)

S2 Fig Rep-like substrate cleavage by TrwC_R_. Denaturing TBE-Urea gels showing the products of cleavage of wt and Rep-like substrates by TrwC_R_. Wt 5’IRDye700-labelled W(25+8) oligonucleotide is shown in lanes 1 and 2, whereas Rep-like 5’IRDye800-labelled H(14+14) oligonucleotide is shown in lanes 3 and 4. Oligonucleotides were incubated with TrwC_R_, treated with proteinase K and SDS, as described in Material and methods. A 25-mer (lane 2) and a 14-mer (lane 4) oligonucleotide appeared in samples treated with TrwC_R_.—represents the control DNA substrate (lanes 1 and 3).
(PDF)

S3 Fig Strand transfer reaction catalyzed by TrwC_R_ with wt, Rep-like and reverse substrates. (A) Schematic representation of the strand transfer reaction. TrwC_R_ cleaves oligonucleotides containing the R388 *nic*-site and performs the strand transfer reaction to a labeled oligonucleotide W(25+0). In green are shown the oligonucleotides that remain covalently attached to TrwC_R_. The gels show the appearance of a labeled transfer product. (B) Denaturing gels showing the strand transfer reaction catalyzed by TrwC_R_ with wt, Rep-like and reverse substrates. 5’IRDye-labelled W(25+0) oligonucleotide is shown in control lanes 1 and 7. A 25+x labeled oligonucleotide was obtained after strand transfer reaction with the wild type oligonucleotides W(25+18), W(18+18), W(14+14) and W(8+8) in lanes 2, 3, 4 and 5, respectively. Strand transfer reaction to W(25+0) was also analyzed for Rep-like H14+x oligonucleotides. Lane 6, H(14+8); lane 8, H(14+10); lane 9, H(14+12); lane 10, H(14+13); lane 11, H(14+14); lane 12, H(14+15) and lane 13, H(14+17). Reverse substrates were also analyzed for strand transfer in lane 14, R(7+27); lane 15, R(8+24); lane 16, R(8+27) and lane 17, R(8+14).
(PDF)

S4 Fig Chromatograms of *nic*-containing oligonucleotides with or without TrwC_R_. 6.3 μM TrwC_R_ was incubated during one hour in presence of EDTA with a 1.5:1 molar excess of each oligonucleotide. Then 20 μl of the samples were injected in a S75 column using the ETHAM system (GE Biosciences). Free oligonucleotides at the same concentration were also injected separately for comparison. Chromatograms of oligonucleotides are shown as dashed lines while TrwC_R_ with oligonucleotides are shown as continuous lines. Several shifted peaks and broaden peaks are observed in the cases of short hairpins H(14+14), H(14+15) and H(14+17). TrwC_R_ interacts with oligonucleotides with longer stems H(16+16), H(23+23), and H(23+26) in a similar fashion. Reverse oligonucleotides R(8+27), R(8+24), R(7+27) show a slight shift when TrwC_R_ is bound to it, similar to the one obtained with wt oligonucleotides (data not shown). Oligonucleotides tested were Rep-like oligonucleotides with D = P = 6 and different loop lengths; H(14+14) S = 8, H(14+15) S = 9 and H(14+17), Rep-like oligonucleotides with S = 11 and different stem lengths: H(16+16) D = P = 8 S = 8, H(23+23) D = P = 15 S = 8, and H(23+26) D = P = 15 S = 11 and Reverse oligonucleotides; R(8+27) D = P = 6 U = 8 S = 11, R(8+24) D = P = 6 U = 8 S = 8 and R(7+27) with D = P = 6 U = 7 S = 11. Absorbance at 260 nm was used during chromatography to determine the presence of DNA. All the chromatograms are normalized and shifted peaks are shown by stars.
(PDF)

S5 Fig Chromatogram and acrylamide native gel analysis of TrwC_R_ with fluorescent oligonucleotide H14+14. 6.3 μM TrwC_R_ was incubated during one hour at RT in presence of 10 mM MgCl_2_ with a 2:1 molar excess of IRDye H(14+14) oligonucleotide. Then 20 μl of the sample was injected in a S75 column using the ETHAM system (GE Biosciences). The chromatogram shows the obtained four A_260_ peaks with the Ve of each peak. After gel filtration column chromatography, 20 μl of 19 fractions collected within these four peaks were loaded into an acrylamide native gel. Lane M contains free oligo. Shifted oligonucleotides are shown by arrowheads. Gels were scanned in Odyssey (LI-COR).
(PDF)

S6 Fig SDS-PAGE gel of TrwC_R_ cleavage reactions on Rep-like substrates with short (L = 6) and long (L = 15/16) loops. 6.3 μM TrwC_R_ was incubated with 15 μM of different oligonucleotides. Lane 1, no oligonucleotide; lane 2, H(14+14) P = D = 6 S = 8; lane 3, H(14+17) P = D = 6 S = 11; lane 4, H(23+23) P = D = 15 S = 8, lane 5, H(23+26) P = D = 15 S = 11, lane 6 H(24+24) P = D = 16 S = 8; lane 7, H(24+27) P = D = 16 S = 11. Lane 8, SDS-low range molecular ladder.
(PDF)

S7 Fig Comparison of relaxases TrwC_R_, TraI_R_ and MobA_R_ used in this study. (A) Location of the catalytic tyrosine and HUH motif in relaxases and replicases. The alignment of Y and HUH motifs in the model relaxases used in this study is shown. (B) Three dimensional structure of HUH relaxases TrwC_R_, TraI_R_ and MobA_R_. 3D structures of TrwC_R_ and MobA_R_ were determined by x-ray crystallography (PDBs 1OMH and 2NS6) while TraI_R_ was modelled with RaptorX (raptorx.uchicago.edu). HUH relaxases have the catalytic tyrosine within an α-helix (depicted in blue in the ribbon structures) and the motif H+HUH located in two juxtaposed β-sheets (depicted in light pink and wheat respectively). (C) SDS PAGE gels showing the protein purity after HPSP (Lane1, TrwC_R_ and TraI_R_) or Heparin (Lane1, MobA_R_) column chromatography and the protein purity after S75 gel filtration column chromatography (lanes 2). M, standards of the Low Range Protein Ladder (BioRad). Overlay of S75 chomatograms of the gel filtration molecular weight markers Bovine-Serum-Albumin (BSA, 67 kDa) and Ribonuclease A (RBA, 13,7 kDa) (blue); TrwC_R_ (green); TraI_R_ (yellow) and MobA_R_ (orange). All the relaxases elute as monomers with an apparent calculated molecular weight of 30 kDa for TrwC_R_ and TraI_R_, and 21 kDa for MobA_R_.
(PDF)

S8 Fig Alignment of the synthetic *oriT* based on Rep and reverse-like *nic* sites. The four inverted repeats (IR_1_ to IR_4_) are highlighted and their sequences underlined. New IR_2_s obtained in Rep-like and reverse plasmids are shown by orange arrows. The *nic* site is indicated with a yellow triangle. Stars show point mutations included in some of the R388 synthetic plasmids to create *BglII*, *BamHI*, *KpnI* and *PstI* restriction sites.
(PDF)
