# Supplementary material for: Design of Novel Relaxase Substrates Based on Rolling Circle Replicases for Bioconjugation to DNA Nanostructures
Source: PLoS One. 2016 Mar 30;11(3):e0152666. doi: 10.1371/journal.pone.0152666 (PMC4814116; doi:10.1371/journal.pone.0152666)
Supplement: S1 Fig — A) Organization of the catalytic centre in Relaxases (TrwCR from R388 plasmid bound to its target (PDB 1OMH)) and RCR replicases (AAV with RBE (PDB 1UUT)). Relaxases and RC-Rep suffered a circular permutation in the primary sequence that localizes the catalytic tyrosine at the N-terminal in the relaxases, but close to the C-terminal in Rep proteins. Relaxases recognize the nic site (red triangle) 5´ to an inverted repeat, while Reps cleave a nic site within a stem-loop. B) Ribbon structure of relaxases (left) and RC-Reps (right). TraIR from plasmid pCU1 (PDB 3L57) and TraIR from plasmid F (PDB 2AOI) belong to MOBF family of relaxases whereas NES from pLW1043 (PDB 4HT4) and minMobA frm R1162 (PDB 2NS6) belong to MOBQ family of relaxases. RepB, the RC initiators of plasmid pMV158 (PDB 3DKX) and Rep from geminivirus (PDB 1L5I) also have a similar folding. All these proteins possess a core of five antiparallel β-strand, where the HUH motif is located at the third β-strand, near the α-helix in which the catalytic tyrosine(s) is held. (PDF) [file pone.0152666.s001.pdf]

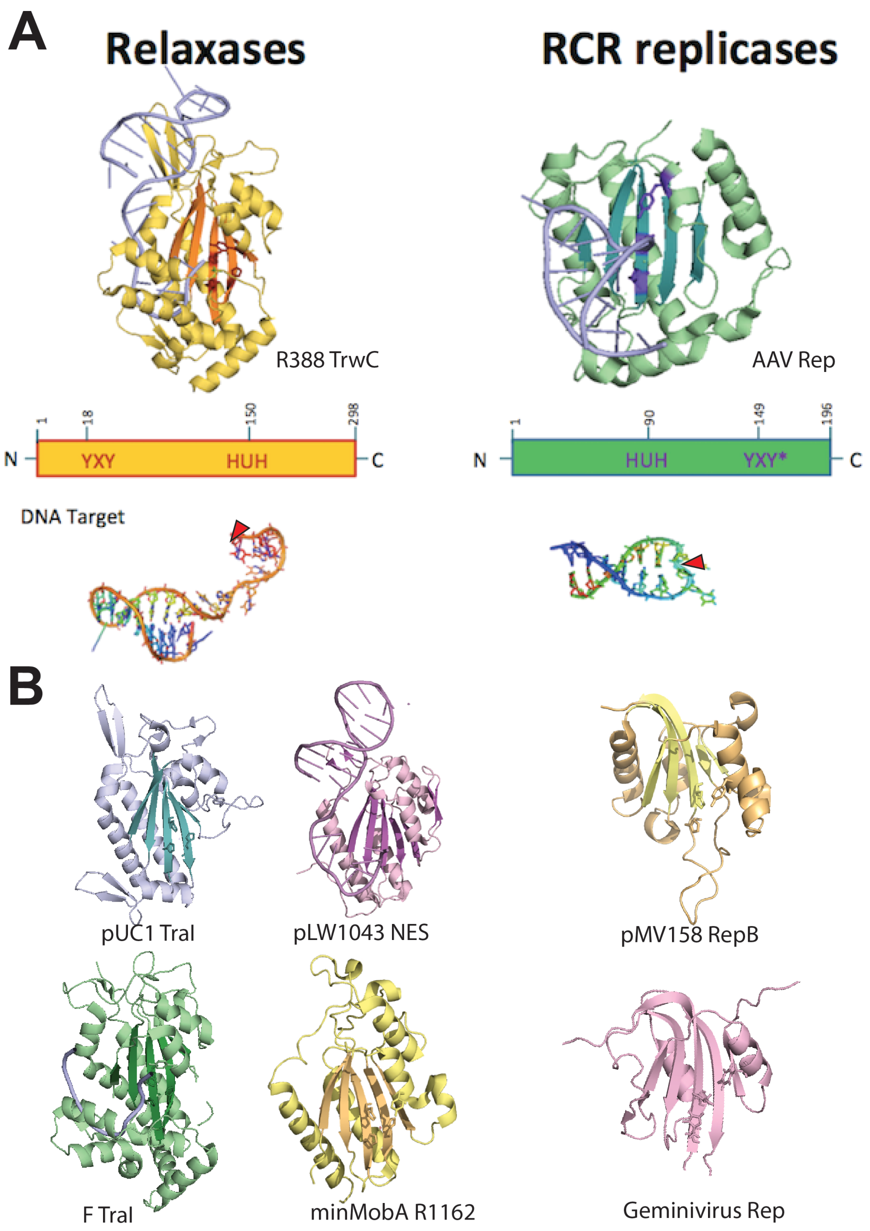

S1 Fig. Comparison of the 3D structure of relaxases and replicases. A) Organization of the catalytic centre in Relaxases ($TrwC_R$ from R388 plasmid bound to its target (PDB 1OMH)) and RCR replicases (AAV with RBE (PDB 1UUT)). Relaxases and RC-Rep suffered a circular permutation in the primary sequence that localizes the catalytic tyrosine at the N-terminal in the relaxases, but close to the C-terminal in Rep proteins. Relaxases recognize the *nic* site (red triangle) 5´to an inverted repeat, while Reps cleave a *nic* site within a stem-loop. B) Ribbon structure of relaxases (left) and RC-Reps (right). $TraI_R$ from plasmid pCU1 (PDB 3L57) and $TraI_R$ from plasmid F (PDB 2AOI) belong to MOB_F family of relaxases whereas NES from pLW1043 (PDB 4HT4) and minMobA frm R1162 (PDB 2NS6) belong to MOB_Q family of relaxases. RepB, the RC initiator of plasmid pMV158 (PDB 3DKX) and Rep from geminivirus (PDB 1L5I) also have a similar folding. All these proteins possess a core of five antiparallel β-strand, where the HUH motif is located at the third β-strand, near the α-helix in which the catalytic tyrosine(s) is held.
