# Supplementary material for: Design of Novel Relaxase Substrates Based on Rolling Circle Replicases for Bioconjugation to DNA Nanostructures
Source: PLoS One. 2016 Mar 30;11(3):e0152666. doi: 10.1371/journal.pone.0152666 (PMC4814116; doi:10.1371/journal.pone.0152666)
Supplement: S2 Fig — Denaturing TBE-Urea gels showing the products of cleavage of wt and Rep-like substrates by TrwCR. Wt 5’IRDye700-labelled W(25+8) oligonucleotide is shown in lanes 1 and 2, whereas Rep-like 5’IRDye800-labelled H(14+14) oligonucleotide is shown in lanes 3 and 4. Oligonucleotides were incubated with TrwCR, treated with proteinase K and SDS, as described in Material and methods. A 25-mer (lane 2) and a 14-mer (lane 4) oligonucleotide appeared in samples treated with TrwCR.—represents the control DNA substrate (lanes 1 and 3). (PDF) [file pone.0152666.s002.pdf]

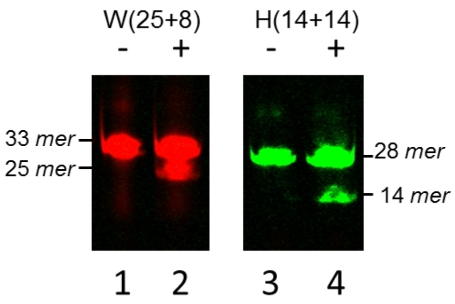

S2.Fig. Rep-like substrate cleavage by $TrwC_R$. Denaturing TBE-Urea gels showing the products of cleavage of wt and Rep-like substrates by $TrwC_R$. Wt 5'IRDye700-labelled W(25+8) oligonucleotide is shown in lanes 1 and 2, whereas Rep-like 5'IRDye800-labelled H(14+14) oligonucleotide is shown in lanes 3 and 4. Oligonucleotides were incubated with $TrwC_R$, treated with proteinase K and SDS, as described in Material and methods. A 25-mer (lane 2) and a 14-mer (lane 4) oligonucleotide appeared in samples treated with $TrwC_R$. - represents the control DNA substrate (lanes 1 and 3).
