# Supplementary material for: Design of Novel Relaxase Substrates Based on Rolling Circle Replicases for Bioconjugation to DNA Nanostructures
Source: PLoS One. 2016 Mar 30;11(3):e0152666. doi: 10.1371/journal.pone.0152666 (PMC4814116; doi:10.1371/journal.pone.0152666)
Supplement: S3 Fig — (A) Schematic representation of the strand transfer reaction. TrwCR cleaves oligonucleotides containing the R388 nic-site and performs the strand transfer reaction to a labeled oligonucleotide W(25+0). In green are shown the oligonucleotides that remain covalently attached to TrwCR. The gels show the appearance of a labeled transfer product. (B) Denaturing gels showing the strand transfer reaction catalyzed by TrwCR with wt, Rep-like and reverse substrates. 5’IRDye-labelled W(25+0) oligonucleotide is shown in control lanes 1 and 7. A 25+x labeled oligonucleotide was obtained after strand transfer reaction with the wild type oligonucleotides W(25+18), W(18+18), W(14+14) and W(8+8) in lanes 2, 3, 4 and 5, respectively. Strand transfer reaction to W(25+0) was also analyzed for Rep-like H14+x oligonucleotides. Lane 6, H(14+8); lane 8, H(14+10); lane 9, H(14+12); lane 10, H(14+13); lane 11, H(14+14); lane 12, H(14+15) and lane 13, H(14+17). Reverse substrates were also analyzed for strand transfer in lane 14, R(7+27); lane 15, R(8+24); lane 16, R(8+27) and lane 17, R(8+14). (PDF) [file pone.0152666.s003.pdf]

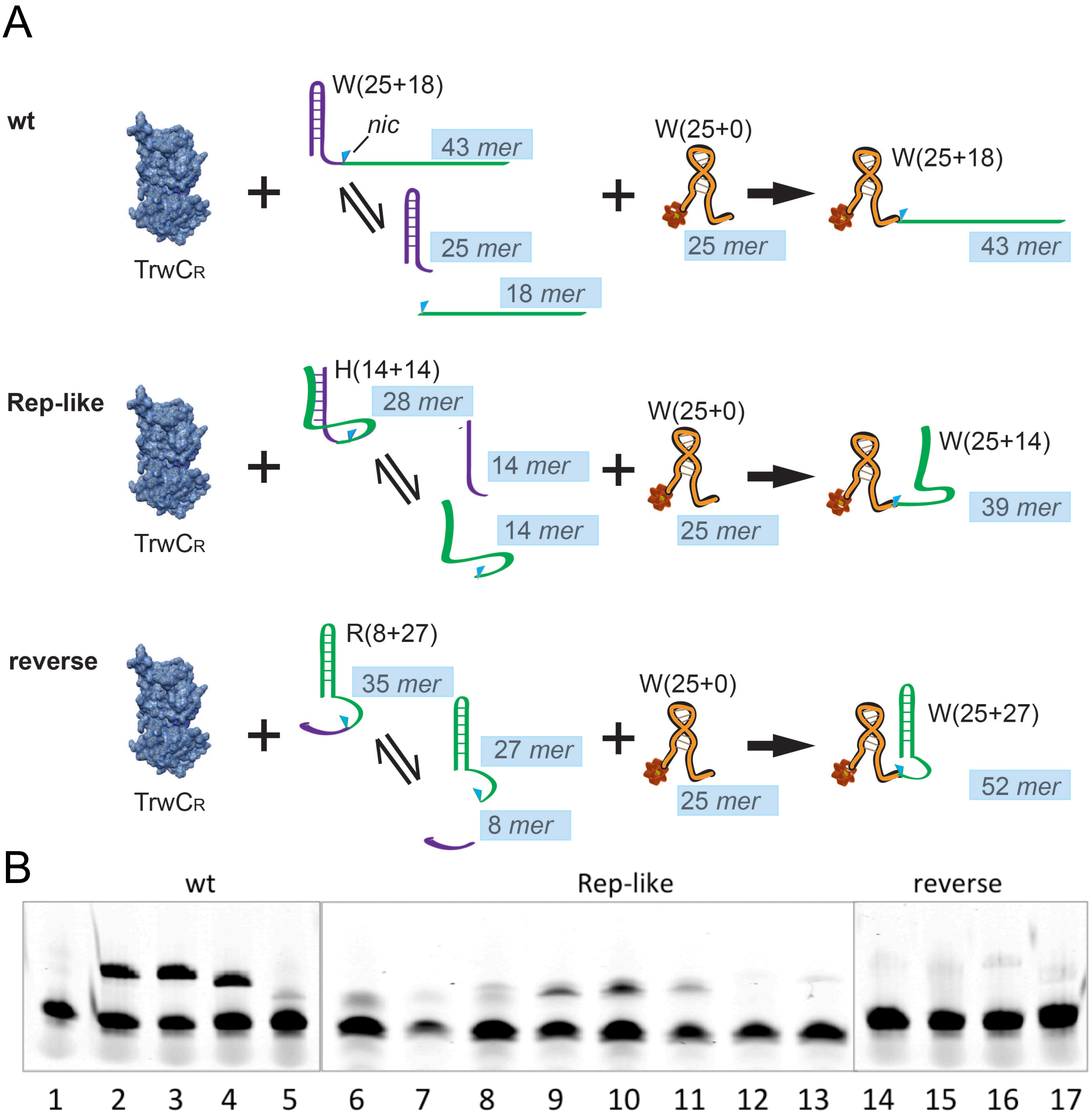

S3 Fig. Strand transfer reaction catalyzed by TrwC$_R$ with wt, Rep-like and reverse substrates. (A) Schematic representation of the strand transfer reaction. TrwC$_R$ cleaves oligonucleotides containing the R388 *nic*-site and performs the strand transfer reaction to a labeled oligonucleotide W(25+0). In green are shown the oligonucleotides that remain covalently attached to TrwC$_R$. The gels show the appearance of a labeled transfer product. (B) Denaturing gels showing the strand transfer reaction catalyzed by TrwC$_R$ with wt, Rep-like and reverse substrates. 5'IRDye-labelled W(25+0) oligonucleotide is shown in control lanes 1 and 7. A 25+x labeled oligonucleotide was obtained after strand transfer reaction with the wild type oligonucleotides W(25+18), W(18+18), W(14+14) and W(8+8) in lanes 2, 3, 4 and 5, respectively. Strand transfer reaction to W(25+0) was also analyzed for Rep-like H14+x oligonucleotides. Lane 6, H(14+8); lane 8, H(14+10); lane 9, H(14+12); lane 10, H(14+13); lane 11, H(14+14); lane 12, H(14+15) and lane 13, H(14+17). Reverse substrates were also analyzed for strand transfer in lane 14, R(7+27); lane 15, R(8+24); lane 16, R(8+27), and lane 17, R(8+14).
