# Supplementary material for: Design of Novel Relaxase Substrates Based on Rolling Circle Replicases for Bioconjugation to DNA Nanostructures
Source: PLoS One. 2016 Mar 30;11(3):e0152666. doi: 10.1371/journal.pone.0152666 (PMC4814116; doi:10.1371/journal.pone.0152666)
Supplement: S4 Fig — 6.3 μM TrwCR was incubated during one hour in presence of EDTA with a 1.5:1 molar excess of each oligonucleotide. Then 20 μl of the samples were injected in a S75 column using the ETHAM system (GE Biosciences). Free oligonucleotides at the same concentration were also injected separately for comparison. Chromatograms of oligonucleotides are shown as dashed lines while TrwCR with oligonucleotides are shown as continuous lines. Several shifted peaks and broaden peaks are observed in the cases of short hairpins H(14+14), H(14+15) and H(14+17). TrwCR interacts with oligonucleotides with longer stems H(16+16), H(23+23), and H(23+26) in a similar fashion. Reverse oligonucleotides R(8+27), R(8+24), R(7+27) show a slight shift when TrwCR is bound to it, similar to the one obtained with wt oligonucleotides (data not shown). Oligonucleotides tested were Rep-like oligonucleotides with D = P = 6 and different loop lengths; H(14+14) S = 8, H(14+15) S = 9 and H(14+17), Rep-like oligonucleotides with S = 11 and different stem lengths: H(16+16) D = P = 8 S = 8, H(23+23) D = P = 15 S = 8, and H(23+26) D = P = 15 S = 11 and Reverse oligonucleotides; R(8+27) D = P = 6 U = 8 S = 11, R(8+24) D = P = 6 U = 8 S = 8 and R(7+27) with D = P = 6 U = 7 S = 11. Absorbance at 260 nm was used during chromatography to determine the presence of DNA. All the chromatograms are normalized and shifted peaks are shown by stars. (PDF) [file pone.0152666.s004.pdf]

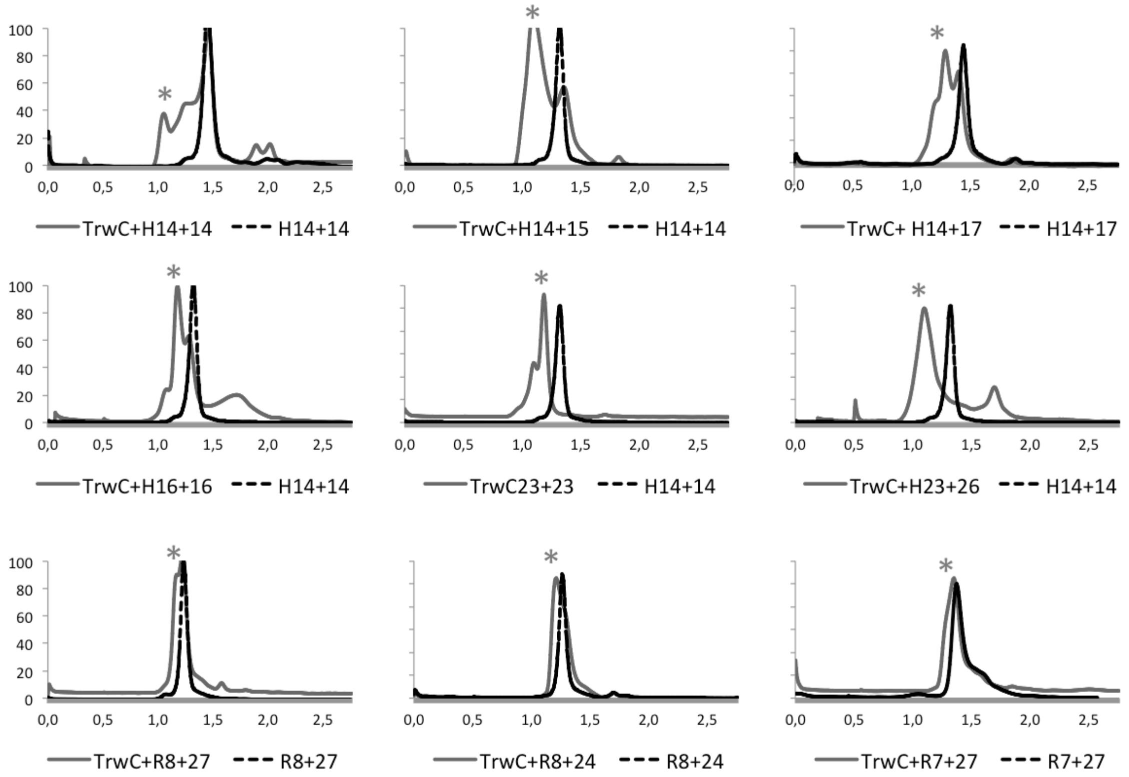

S4 Fig. Chromatograms of *nic*-containing oligonucleotides with or without $TrwC_R$. 6.3 μM $TrwC_R$ was incubated during one hour in presence of EDTA with a 1.5:1 molar excess of each oligonucleotide. Then 20 μl of the samples were injected in a S75 column using the ETHAM system (GE Biosciences). Free oligonucleotides at the same concentration were also injected separately for comparison. Chromatograms of oligonucleotides are shown as dashed lines while $TrwC_R$ with oligonucleotides are shown as continuous lines.. Several shifted peaks and broaden peaks are observed in the cases of short hairpins H(14+14), H(14+15) and H(14+17). $TrwC_R$ interacts with oligonucleotides with longer stems H(16+16), H(23+23), and H(23+26) in a similar fashion. Reverse oligonucleotides R(8+27), R(8+24), R(7+27) show a slight shift when $TrwC_R$ is bound to it, similar to the one obtained with wt oligonucleotides (data not shown). Oligonucleotides tested were Rep-like oligonucleotides with D=P=6 and different loop lengths; H(14+14) S=8 , H(14+15) S=9 and H(14+17), Rep-like oligonucleotides with S=11 and different stem lengths: H(16+16) D=P=8 S=8, H(23+23) D=P=15 S=8, and H(23+26) D=P=15 S=11 and Reverse oligonucleotides; R(8+27) D=P=6 U=8 S= 11, R(8+24) D=P=6 U=8 S= 8 and R(7+27)with D=P=6 U= 7 S= 11. Absorbance at 260 nm was used during chromatography to determine the presence of DNA. All the chromatograms are normalized and shifted peaks are shown by stars.
