# Supplementary material for: Design of Novel Relaxase Substrates Based on Rolling Circle Replicases for Bioconjugation to DNA Nanostructures
Source: PLoS One. 2016 Mar 30;11(3):e0152666. doi: 10.1371/journal.pone.0152666 (PMC4814116; doi:10.1371/journal.pone.0152666)
Supplement: S5 Fig — 6.3 μM TrwCR was incubated during one hour at RT in presence of 10 mM MgCl2 with a 2:1 molar excess of IRDye H(14+14) oligonucleotide. Then 20 μl of the sample was injected in a S75 column using the ETHAM system (GE Biosciences). The chromatogram shows the obtained four A260 peaks with the Ve of each peak. After gel filtration column chromatography, 20 μl of 19 fractions collected within these four peaks were loaded into an acrylamide native gel. Lane M contains free oligo. Shifted oligonucleotides are shown by arrowheads. Gels were scanned in Odyssey (LI-COR). (PDF) [file pone.0152666.s005.pdf]

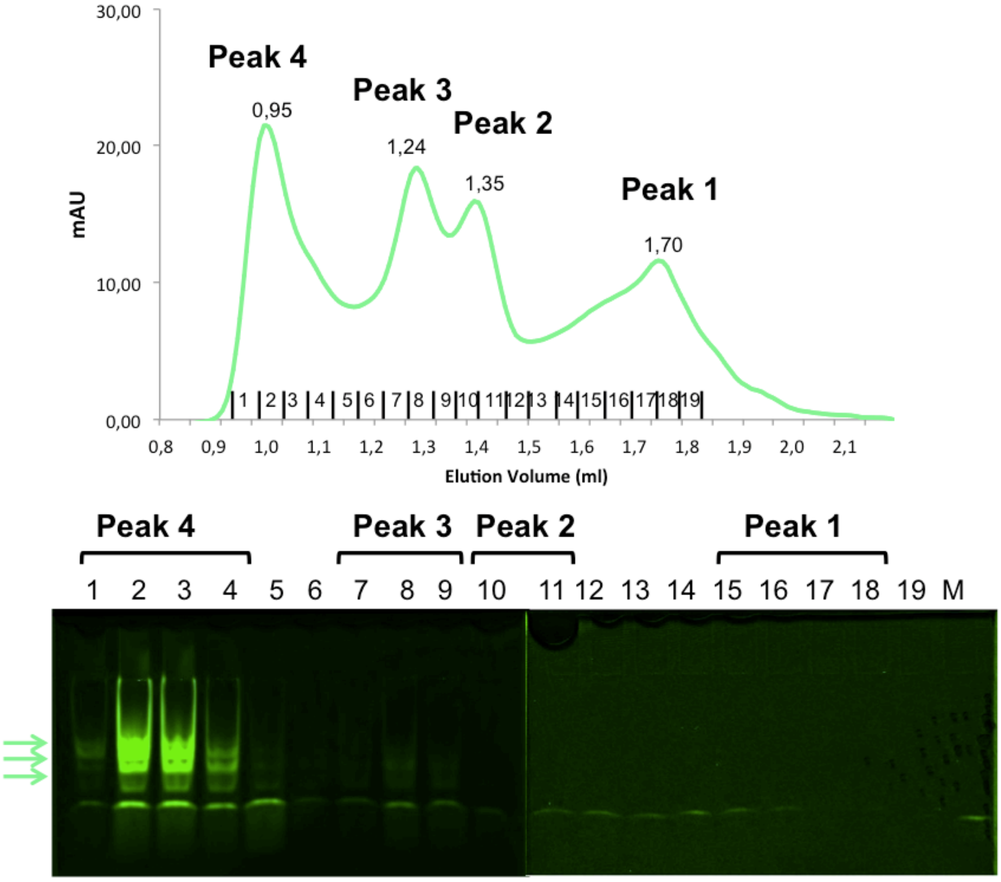

S5 Fig. Chromatogram and acrylamide native gel analysis of $TrwC_R$ with fluorescent oligonucleotide H14+14. 6.3 μM $TrwC_R$ was incubated during one hour at RT in presence of 10 mM $MgCl_2$ with a 2:1 molar excess of IRDye H(14+14) oligonucleotide. Then 20 μl of the sample was injected in a S75 column using the ETHAM system (GE Biosciences). The chromatogram shows the obtained four A_{260} peaks with the Ve of each peak. After gel filtration column chromatography, 20 μl of 19 fractions collected within these four peaks were loaded into an acrylamide native gel. Lane M contains free oligo. Shifted oligonucleotides are shown by arrowheads. Gels were scanned in Odyssey (LI-COR).
