# Supplementary material for: Design of Novel Relaxase Substrates Based on Rolling Circle Replicases for Bioconjugation to DNA Nanostructures
Source: PLoS One. 2016 Mar 30;11(3):e0152666. doi: 10.1371/journal.pone.0152666 (PMC4814116; doi:10.1371/journal.pone.0152666)
Supplement: S6 Fig — 6.3 μM TrwCR was incubated with 15 μM of different oligonucleotides. Lane 1, no oligonucleotide; lane 2, H(14+14) P = D = 6 S = 8; lane 3, H(14+17) P = D = 6 S = 11; lane 4, H(23+23) P = D = 15 S = 8, lane 5, H(23+26) P = D = 15 S = 11, lane 6 H(24+24) P = D = 16 S = 8; lane 7, H(24+27) P = D = 16 S = 11. Lane 8, SDS-low range molecular ladder. (PDF) [file pone.0152666.s006.pdf]

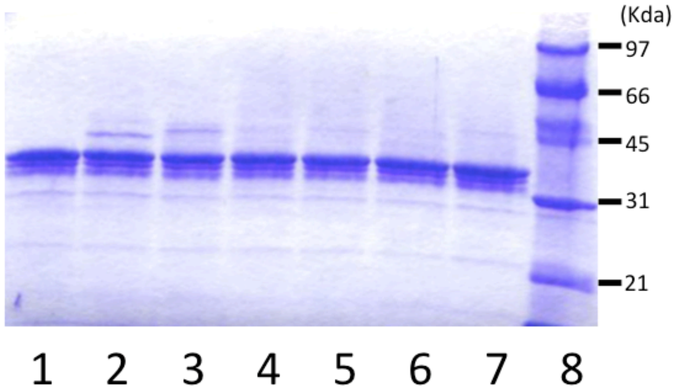


**S6 Fig. SDS-PAGE gel of $TrwC_R$ cleavage reactions on Rep-like substrates with short (L=6) and long (L=15/16) loops.** 6.3 µM $TrwC_R$ was incubated with 15 µM of different oligonucleotides. Lane 1, no oligonucleotide; lane 2, H(14+14) P=D=6 S=8 ; lane 3, H(14+17) P=D=6 S=11; lane 4, H(23+23) P=D=15 S=8, lane 5, H(23+26) P=D=15 S=11, lane 6 H(24+24) P=D=16 S=8; lane 7, H(24+27) P=D=16 S=11. Lane 8, SDS-low range molecular ladder.
