# Supplementary material for: Design of Novel Relaxase Substrates Based on Rolling Circle Replicases for Bioconjugation to DNA Nanostructures
Source: PLoS One. 2016 Mar 30;11(3):e0152666. doi: 10.1371/journal.pone.0152666 (PMC4814116; doi:10.1371/journal.pone.0152666)
Supplement: S7 Fig — (A) Location of the catalytic tyrosine and HUH motif in relaxases and replicases. The alignment of Y and HUH motifs in the model relaxases used in this study is shown. (B) Three dimensional structure of HUH relaxases TrwCR, TraIR and MobAR. 3D structures of TrwCR and MobAR were determined by x-ray crystallography (PDBs 1OMH and 2NS6) while TraIR was modelled with RaptorX (raptorx.uchicago.edu). HUH relaxases have the catalytic tyrosine within an α-helix (depicted in blue in the ribbon structures) and the motif H+HUH located in two juxtaposed β-sheets (depicted in light pink and wheat respectively). (C) SDS PAGE gels showing the protein purity after HPSP (Lane1, TrwCR and TraIR) or Heparin (Lane1, MobAR) column chromatography and the protein purity after S75 gel filtration column chromatography (lanes 2). M, standards of the Low Range Protein Ladder (BioRad). Overlay of S75 chomatograms of the gel filtration molecular weight markers Bovine-Serum-Albumin (BSA, 67 kDa) and Ribonuclease A (RBA, 13,7 kDa) (blue); TrwCR (green); TraIR (yellow) and MobAR (orange). All the relaxases elute as monomers with an apparent calculated molecular weight of 30 kDa for TrwCR and TraIR, and 21 kDa for MobAR. (PDF) [file pone.0152666.s007.pdf]

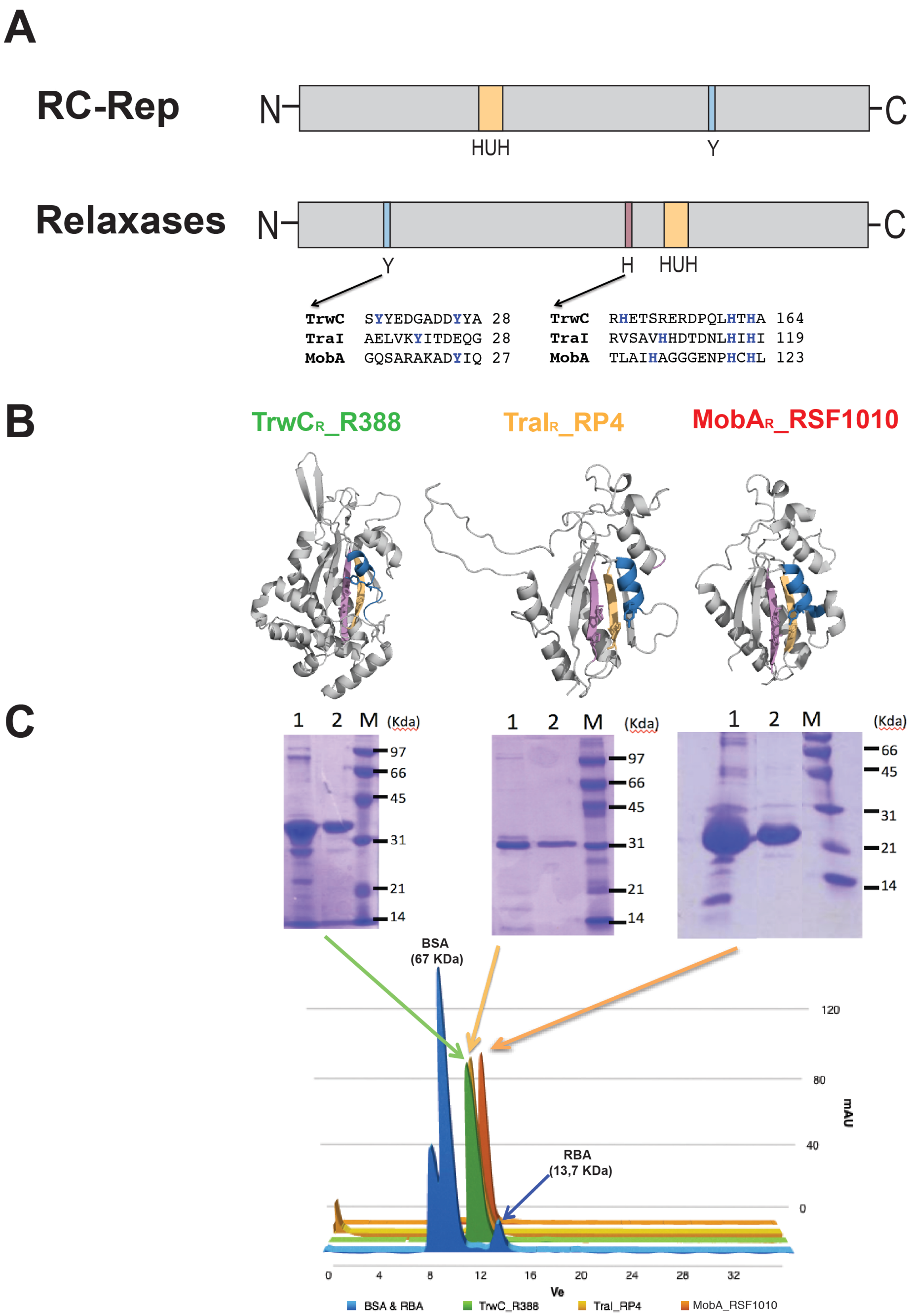

S7 Fig. Comparison of relaxases TrwC$_R$, TraI$_R$ and MobA$_R$ used in this study. (A) Location of the catalytic tyrosine and HUH motif in relaxases and replicases. The alignment of Y and HUH motifs in the model relaxases used in this study is shown. (B) Three dimensional structure of HUH relaxases TrwC$_R$, TraI$_R$ and MobA$_R$. 3D structures of TrwC$_R$ and MobA$_R$ were determined by x-ray crystallography (PDBs 1OMH and 2NS6) while TraI$_R$ was modelled with RaptorX (raptorx.uchicago.edu). HUH relaxases have the catalytic tyrosine within an α-helix (depicted in blue in the ribbon structures) and the motif H+HUH located in two juxtaposed β-sheets (depicted in light pink and wheat respectively). (C) SDS PAGE gels showing the protein purity after HPSP (Lane1, TrwC$_R$ and TraI$_R$) or Heparin (Lane1, MobA$_R$) column chromatography and the protein purity after S75 gel filtration column chromatography (lanes 2). M, standards of the Low Range Protein Ladder (BioRad). Overlay of S75 chomatograms of the gel filtration molecular weight markers Bovine-Serum-Albumin (BSA, 67 kDa) and Ribonuclease A (RBA, 13,7 kDa) (blue); TrwC$_R$ (green); TraI$_R$ (yellow) and MobA$_R$ (orange). All the relaxases elute as monomers with an apparent calculated molecular weight of 30 kDa for TrwC$_R$ and TraI$_R$, and 21 kDa for MobA$_R$.
