# Supplementary material for: Design of Novel Relaxase Substrates Based on Rolling Circle Replicases for Bioconjugation to DNA Nanostructures
Source: PLoS One. 2016 Mar 30;11(3):e0152666. doi: 10.1371/journal.pone.0152666 (PMC4814116; doi:10.1371/journal.pone.0152666)
Supplement: S8 Fig — The four inverted repeats (IR1 to IR4) are highlighted and their sequences underlined. New IR2s obtained in Rep-like and reverse plasmids are shown by orange arrows. The nic site is indicated with a yellow triangle. Stars show point mutations included in some of the R388 synthetic plasmids to create BglII, BamHI, KpnI and PstI restriction sites. (PDF) [file pone.0152666.s008.pdf]

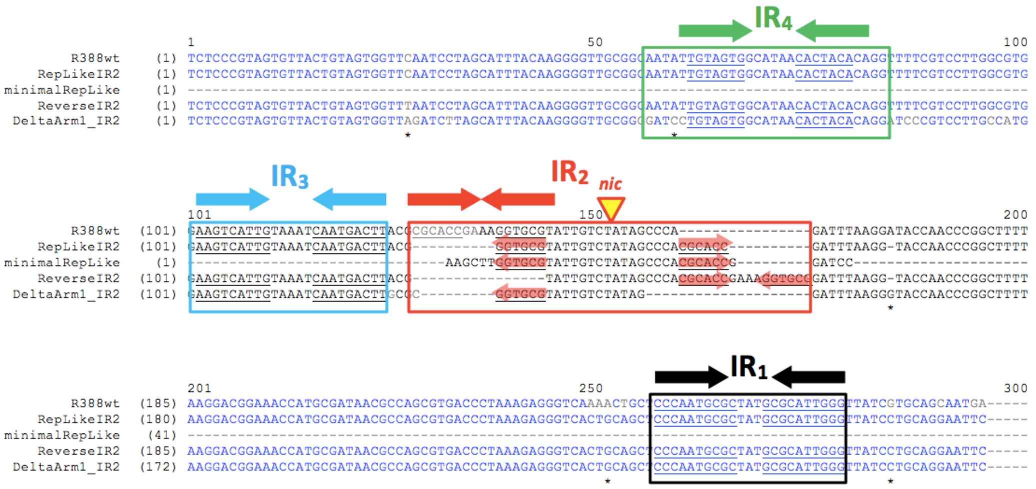

S8 Fig. Alignment of the synthetic *oriT* based on Rep and reverse-like *nic* sites. The four inverted repeats (IR_1 to IR_4) are highlighted and their sequences underlined. New IR_2s obtained in Rep-like and reverse plasmids are shown by orange arrows. The *nic* site is indicated with a yellow triangle. Stars show point mutations included in some of the R388 synthetic plasmids to create *BglII, BamHI, KpnI* and*PstI* restriction sites.
